# LIPUS promotes calcium oscillations to improve lymphatic drainage and alleviates cold-stimulated pain and effusion in osteoarthritis joints

**DOI:** 10.1016/j.jot.2026.101242

**Published:** 2026-10-24

**Authors:** Jun Yang, Renhao Liu, Xin Yang, Xiaohong Li, Xiuqin Peng, Jiaming Han, Peidong Pu, Jun Liu, Yu Tian, Song Li, Junmei Qi, Hangang Chen, Jing Yang, Siru Zhou, Liang Chen, Nan Su, Ying Zhu, Yangli Xie, Liang Kuang, Lin Chen

**Affiliations:** a Center of Bone Metabolism and Repair, Laboratory for Prevention and Rehabilitation of Military Training Injuries, State Key Laboratory of Trauma and Chemical Poisoning, Trauma Center, Research Institute of Surgery, Daping Hospital, Army Medical University, Chongqing, 400042, China; b Collaborative Innovation Centre of Regenerative Medicine and Medical BioResource Development and Application Co-constructed by the Province and Ministry, Guangxi Medical University, Nanning, Guangxi, 530021, China; c Department of Rehabilitation Medicine, Key Laboratory of Physical Medicine and Precision Rehabilitation of Chongqing Municipal Health Commission, The First Affiliated Hospital of Chongqing Medical University, Chongqing, 400010, China; d War Trauma Medical Center, State Key Laboratory of Trauma and Chemical Poisoning, Army Medical Center, Daping Hospital, Army Medical University, Chongqing, 40038, China

**Keywords:** Calcium oscillation, Cold exposure, LIPUS, Lymphatic drainage, Osteoarthritis, TRPV4

## Abstract

**Background:**

Osteoarthritis (OA) patients in remission usually experience acute exacerbations of pain and joint swelling following cold stimulation. This represents one of the characteristic clinical presentations of “cold knees”. In Traditional Chinese Medicine, this phenomenon correlates with “Cold Bi”, reflecting “lingering dampness” and “stagnation of Blood and Qi” in the joints, and is conceptually consistent with dysfunction of synovial lymphatic system (SLS) observed in OA. However, the underlying mechanisms of cold-exacerbated OA and the therapeutic strategies remain poorly explored.

**Methods:**

OA mouse models were established by destabilization of the medial meniscus (DMM) surgery. The mice were exposed to cold stimulation at 4 weeks post-surgery to mimic OA patients in remission experiencing acute cold stimulation. Joint pain-related behaviors were assessed using multiple approaches including gait analysis and von Frey test. Magnetic Resonance Imaging (MRI) was used to evaluate joint effusion. Near-infrared indocyanine green (NIR-ICG) imaging and intra-articular injections of Fluorescein isothiocyanate-dextran (FITC-dextran) were employed to assess lymphatic drainage. Furthermore, the effects of temperature decrease and low-intensity pulsed ultrasound (LIPUS) on calcium oscillations and nitric oxide (NO) production by lymphatic endothelial cells (LECs) were investigated using live-cell fluorescence imaging. Additionally, the mechanisms through which LIPUS plays therapeutic roles were also explored using transient receptor potential vanilloid 4 **(**TRPV4) inhibitor HC-067047.

**Results:**

Acute cold stimulation aggravated gait abnormality and decreased paw withdrawal threshold in DMM-operated mice. Furthermore, weight-bearing asymmetry and total locomotor distance in the open field were also worsened in these mice. The knee joint effusion was significantly aggravated following acute cold stimulation accompanied by impaired synovial lymphatic drainage. Mechanistically, cold stimulation suppressed calcium oscillations of LECs and decreased their NO production, which were efficiently restored by LIPUS. Additionally, LIPUS treatment improved lymphatic drainage of the knee joint and alleviated cold-stimulated exacerbation of OA phenotypes in mice. These therapeutic effects were significantly suppressed under TRPV4 inhibition as well as hindlimb lymphatic vessel ligation.

**Conclusion:**

Dysfunction of lymphatic drainage contributes to cold-stimulated exacerbation of OA phenotypes in mice, which could be alleviated by LIPUS-induced NO production through the TRPV4/Ca^2+^/eNOS/NO axis. These findings provide novel insights into the mechanisms of “cold knees” and highlight the therapeutic potential of LIPUS in the treatment of pain and joint effusion in OA.

The translational potential of this article: Our study suggests that LIPUS ameliorates cold-induced worsening of pain and joint effusion in OA by improving synovial lymphatic drainage. These findings provide preliminary evidence for the translational potential of LIPUS application in skeletal disorders characterized by lymphatic drainage dysfunction.

## Graphical abstract

**Figure:**
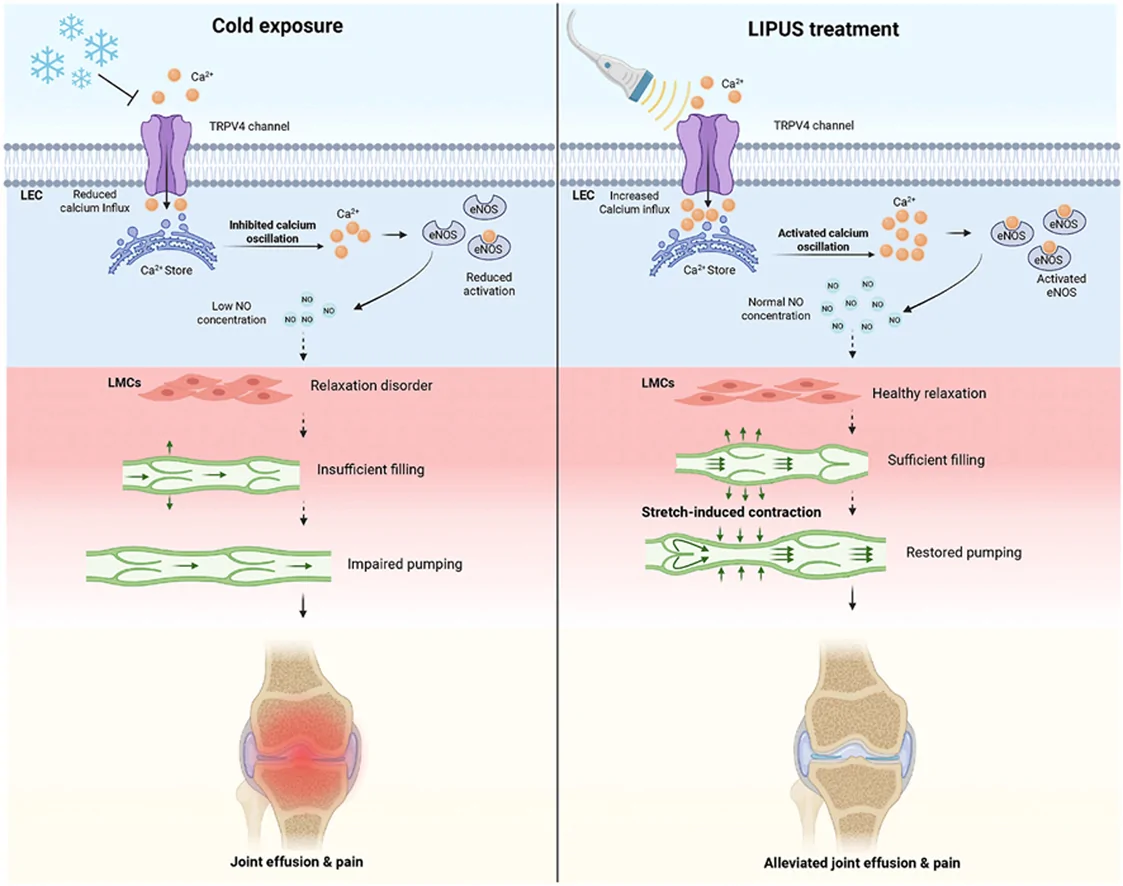

## 1 Introduction

Osteoarthritis (OA) is one of the most common joint diseases, characterized by joint pain, swelling, and impaired mobility. It affects approximately 528 million people worldwide [1]. As the predominant symptom, joint pain is a significant reason for the patients to seek medical attention. An animal study has demonstrated that prolonged low-temperature exposure exacerbates OA pathogenesis such as cartilage degeneration [2]. In Traditional Chinese Medicine, arthritis is referred to as “Bi syndrome”. Among its types, “Cold Bi” describes joints and muscle tissues experiencing “lingering dampness” and “stagnation of blood and Qi” after cold stimulation. Furthermore, low temperatures compromise the clearance of pathogenic factors, leading to joint pain and swelling [3]. This represents a well-recognized clinical phenomenon in which OA patients in remission experience exacerbation of pain and joint swelling following acute cold stimulation [4], [5], which is consistent with the characteristic presentation of “cold knees” [6].

In modern histological and physiological principles, lymphatic vessels contribute to draining metabolic waste, inflammatory substances, and cells from tissues [7]. Substances within the joint cavity enter lymphatic vessels through synovial lymphatic stomata [8], proceeding into capillary lymphatics and then into collecting lymphatics. Driven by the spontaneous contraction of lymphatic muscle cells (LMCs) surrounding the collecting lymphatics and extrinsic mechanical forces from nearby skeletal muscles [9], the lymph is further pumped to draining lymph nodes (DLNs), specifically, the iliac lymph nodes (ILNs) for the knee joint, and eventually returns to the circulation via the thoracic duct [10]. The function of the synovial lymphatic system (SLS) directly determines the stability of the synovial immune microenvironment, subsequently influencing articular cartilage homeostasis through tissue crosstalk [11]. In active OA, joint effusion is closely associated with synovitis and joint pain [12]. The accumulation of immune cells, inflammatory factors, algogenic substances, and matrix-degrading enzymes exacerbates joint pain, which is closely related to SLS dysfunction [10], [13]. Furthermore, lower temperature inhibits lymphatic contraction and dampens lymphatic drainage [14], providing a potential mechanism for the “cold knees” observed in OA patients. Nevertheless, the role of lymphatic drainage in cold-induced acute exacerbation of OA and the corresponding therapeutic strategies remain largely unexplored.

Low-intensity pulsed ultrasound (LIPUS) is a safe and non-invasive physical therapy modality which acts predominantly through mechanical rather than thermal bioeffects [15], [16]. It activates various mechanosensitive ion channels, predominantly the calcium channels [17]. Numerous studies have elucidated the roles and mechanisms of LIPUS in OA treatment [18]. For instance, LIPUS delays cartilage degeneration by restoring chondrocytes autophagy and inhibiting the NF-κB signaling [19]. It also inhibits synovial fibroblast proliferation and reduces synovial fibrosis [20]. More recently, we reveal that the autophagy-promoting and chondroprotective effects of LIPUS are regulated by transient receptor potential vanilloid 4 (TRPV4) mediated calcium oscillations in chondrocytes [18]. Furthermore, LIPUS was also found to promote the clearance of waste from brain parenchyma through lymphatic drainage [21]. However, whether LIPUS could be used to promote synovial lymphatic drainage and alleviate joint pain or effusion in “cold knees” remains unclear.

In this study, we hypothesize that acute cold stimulation exacerbates OA pain and joint effusion by impairing lymphatic drainage. We established an acute cold-stimulation model in mice at 4 weeks post-DMM (destabilization of the medial meniscus) surgery, a stage characterized by moderate synovitis and mild pain [22], to mimic cold-induced acute exacerbation in OA patients in remission. We observed that acute cold stimulation significantly aggravated pain and joint effusion in OA mice, concurrent with significantly impaired lymphatic drainage. Furthermore, our data suggested that low temperature inhibited calcium oscillations in lymphatic endothelial cells (LECs), which contributed to lymphatic contractile dysfunction in “cold knees”. Additionally, LIPUS treatment ameliorated lymphatic drainage dysfunction and alleviated cold-stimulated pain and effusion in OA mice. These effects could be mediated by the restoration of TRPV4-dependent calcium oscillations and the subsequent NO production in LECs. Collectively, our study reveals the critical roles of the synovial lymphatic system in the pathogenesis of “cold knees” and highlights the therapeutic potential of LIPUS in improving joint lymphatic drainage and alleviating OA-related symptoms such as joint pain and effusion.

## 2 Materials and methods

### 2.1 Animal experiments

Following a previously described protocol, destabilization of the medial meniscus **(**DMM) surgery was performed on the right knee joints of 12-week-old C57BL/6J male mice from Charles River Laboratory Animal Technology (Beijing, China) to induce knee OA. Briefly, the joint cavity of an anesthetized mouse was exposed after an incision in the patellar ligament, and then the medial meniscotibial ligament (MMTL) was severed using a microsurgical scalpel [22]. As a control, the sham operation was performed by incision of the patellar ligament only. Mice were randomly divided into groups and treated as indicated. For cold stimulation, mice were placed in a 4 °C incubator for 12 h every day for a total of 3 consecutive days. During the cold exposure, the animals were closely monitored for their general condition and signs of distress, including changes in body weight, posture, locomotor activity, grooming status/piloerection, food and water intake, as well as abnormal lethargy or impaired mobility. Humane endpoint criteria were established in accordance with institutional guidelines, and any animal showing severe or persistent distress would be removed from the study and euthanized if necessary.

### 2.2 LIPUS treatment

LIPUS treatment began one day after cold stimulation and continued for three consecutive days. Briefly, the anesthetized mice were shaved and then placed in the prone position, with the medial side of the operated knee facing downward and positioned directly over the upward-facing LIPUS probe. A layer of ultrasound coupling gel approximately 1 mm thick was applied between the skin over the knee joint and the probe. The parameters, ultrasound frequency 1.5 MHz, pulse repetition frequency 1.0 kHz, intensity 30 mW/ ${\text{cm}}^{2}$, duty cycle 20%, duration 20 min, were adopted as previously reported [20]. These combinations represent classic, safe, and previously validated parameters for osteoarthritis treatment [23], which provide sufficient mechanical stimulation while minimizing the potential risk of thermal effects and exerting multiple bioeffects such as anti-inflammation [24], calcium signaling activation and chondroprotective effects [18]. Furthermore, similar parameters were used for LIPUS-induced lymphatic-promoting therapies [25], [26]. For mice without LIPUS treatment, the same preparing procedures were performed on mice with LIPUS powered off. The LIPUS device was generously supplied by Prof. Xiaolan Du.

### 2.3 Lymphatic drainage blockade

The surgical procedure for lymphatic drainage blockade was performed as described previously [27]. The mice were anesthetized and Evans blue was injected intra-articularly to visualize the inguinal lymphatic vessels. Incisions of about 1 cm were made to expose the inguinal regions of the mice using surgical scissors. Then, the Evans blue-stained lymphatic vessels were cut and ligated with 10-0 nylon thread.

### 2.4 Behavioral testing

Pain-related behavior of mice was assessed by hind limb weight distribution, open field tests, and gait analysis [28]. Hind limb weight-bearing balance was tested by a static weight-bearing device (BIO-SWB-TOUCH/R, Bioseb). The weight-bearing asymmetry was calculated as difference between hind limbs as a percentage of total weight borne through both hind limbs [29]. For the open field test, mice were allowed to move freely for 5 min, and their locomotor activity was tracked using video analysis. Gait data were subsequently quantified using the CatWalk XT 10 gait analysis system (Noldus, Netherlands). von Frey test was performed to calculate the withdrawal threshold (BIO-ECF4, Bioseb).

### 2.5 Magnetic resonance imaging and joint effusion assessment

Magnetic resonance imaging (MRI) of the knee joint in the cold stimulation group was performed using a 7.0T Bruker (Ettlingen, Germany) BioSpec 70/20USR magnetic resonance microimaging system (Department of Radiology, Daping Hospital, Army Medical University, Chongqing, China). Joint effusion was defined as areas of hyperintensity on T2-weighted images. Quantification of joint effusion volume and 3D reconstruction were performed with 3D Slicer software 5.2.1.

### 2.6 Histopathological staining

Fresh mouse knee joints were fixed in 4% paraformaldehyde solution for 24 h, and subsequently decalcified in 0.5 M EDTA solution for 2 weeks. The knee joints were embedded in paraffin and serially sectioned at a thickness of 5 μm and the sections were selected from medial to lateral compartments at approximately 50 μm intervals. Eight to ten representative mid-sagittal sections were selected and deparaffinized in xylene, then rehydrated through a graded ethanol series. Safranin-O/fast green and Hematoxylin and Eosin **(**H&E) staining were used to evaluate cartilage destruction and synovitis respectively. Degeneration of articular cartilage was quantified by independent observers using the Osteoarthritis Research Society International (OARSI) scoring system in a blinded manner [30]. The synovitis score was performed using methods described by previous research [31].

For immunofluorescence staining, paraffin-embedded knee joint sections were rehydrated through a graded ethanol series and rinsed in phosphate-buffered saline (PBS). After antigen retrieval, sections were blocked with blocking buffer (QuickBlock, Beyotime, Shanghai, China) for 1 h at room temperature and incubated with primary antibodies (anti-F4/80, Abcam, ab6640) overnight at 4 °C. After washing with PBS, sections were incubated with appropriate fluorophore-conjugated secondary antibodies for 1 h at 37 °C in the dark. Nuclei were counterstained with DAPI, and the sections were mounted with an antifade mounting medium. Fluorescence images were acquired using a fluorescence microscope.

### 2.7 Lymphatic drainage assessment

For near-infrared indocyanine green (NIR-ICG) imaging, ICG was dissolved in saline with a concentration of 0.1 mg/mL, and 5 μL ICG solution was injected intra-articularly. Subsequently, ICG images of mouse knee joints were obtained by Fusion FX Edge system (Vilber Lourmat). The signal intensity in the knee was recorded immediately after ICG injection as the initial signal intensity and ICG image was captured again 6 h after ICG injection. The images were analyzed using ImageJ software. Regions of interest (ROI) covering the injection site of the knee were defined. Synovial lymphatic drainage, as indicated by the percentage of ICG clearance, was calculated based on the decay in ICG signal intensity between the 0-h and 6-h images from the ROI in the knee joint after ICG injection [32].

For FITC-dextran lymphatic imaging, we selected 250 kDa dextran based on evidence that synovial lymphatics preferentially absorb macromolecules larger than 40 kDa [11]. Moreover, as previously validated by Lin et al. [33], this tracer colocalizes exclusively with lymphatic vessels rather than blood vessels. Mice were injected with FITC-dextran (250 kDa, FD250S, Sigma) intra-articularly 6 h before sacrifice. Then knee joints and ILNs draining the knee were harvested and fixed in 4% paraformaldehyde solution for 24 h, and knee joints were decalcified in 0.5 M EDTA solution for 2 weeks. The knee joints and ILNs were embedded in OCT (Thermo) and sectioned at a thickness of 16 μm using a cryostat (Leica). For synovial influx, frozen sections of knee joints were washed with PBS to remove free dextran molecules from the joint cavity, and imaged with fluorescence microscope (Olympus). The number of dextran particles in the synovial sublining layer was quantified by Image J software to represent synovial influx. For DLN capacity, frozen sections of ILN were imaged with fluorescence microscope (Olympus) and the number of dextran particles in the ILN was quantified as DLN capacity using Image J software [33].

### 2.8 LECs culture

The mouse lymphatic endothelial cell line, SVEC4-10 (CL-0221, Procell, Wuhan, China) was used. SVEC4-10 were cultured in SVEC4-10 Cell Complete Medium (CM-0221, Procell, Wuhan, China). SVEC4-10 were subjected to cold exposure by incubation at 33 °C for 30 min. For TRPV4 blockade, SVEC4-10 were pre-treated with HC-067047 (S6637, Selleck) for 30 min. Cellular proteins and RNA were harvested 30 min after LIPUS treatment.

### 2.9 Lymphatic endothelial cells calcium oscillation fluorescence imaging

To evaluate the changes in intracellular calcium concentrations, a calcium-sensitive fluorescence indicator, Fluo-4 AM Calcium Assay Kit (S1061S, Beyotime), was used to label LECs. As previously described, confluent 35-mm glass-bottom microwell dishes containing cells were first washed 3 times in HBSS without Ca^2+^ and labeled with 4 μM Fluo-4 AM diluted in assay buffer in the kit at 37 °C in 5% CO_2_ for approximately 30 min. Shielded from the light, the dishes were washed 3 times with HBSS and then phenol red-free DMEM was added. Next, the cells were visualized and imaged to observe calcium oscillations using an Olympus IX81 fluorescence microscope (Olympus Optical, Japan) under different temperature conditions with the LIPUS treatment (parameters: ultrasound frequency 1.5 MHz; pulse repetition frequency 1.0 kHz; intensity 30 mW/cm^2^; duty cycle 20%) as shown in Fig. 5A. Eight cells were randomly selected to plot the curves of calcium transient relative fluorescence intensity.

ImageJ software was used to analyze each ROI. Signal intensity was presented as relative fluorescence changes (ΔF/F) after background subtraction. A calcium peak was identified as a transient increase of Ca^2+^ signal intensity exceeding 1.25 times the baseline intensity of the cell. The total number of peaks during recording in each cell was counted. Cells exhibiting at least one calcium peak were classified as responsive cells. The calcium peak magnitude was reported as the mean fold change of the peak intensity over the mean baseline fluorescence intensity in ROI. The responsive rate was calculated as the ratio of responsive cells to total cells in the field of view [34].

### 2.10 Western blotting

Proteins were extracted from SVEC4-10 using RIPA lysis buffer (P0013B, Beyotime) containing protease inhibitors. The protein samples were subjected to electrophoresis on 10% sodium dodecyl sulfate-polyacrylamide gels and subsequently transferred to PVDF membranes (IPVH00010, Millipore). The membranes were then blocked with TBST containing 8% skim milk, followed by overnight incubation at 4 °C with the appropriate primary antibodies (anti-p-eNOS, ab215717, Abcam; anti-eNOS, ab199956, Abcam). Secondary anti-rabbit antibodies were applied for 1 h at 37 °C. After washing with TBST, the signal was detected using ChemiScope 6000 (Clinx Science Instruments, China).

### 2.11 Quantitative real-time PCR

Total RNA was isolated from the treated SVEC4-10 using TRIzol™ reagent (15596026, Invitrogen). The RNA samples were subsequently reverse-transcribed and subjected to quantitative PCR according to the manufacturer's instructions for the PrimeScript™ RT Kit (RR047A, Takara) and the SYBR Premix Ex Taq™ Kit (RR820A, Takara). Amplification was performed on a Mx3000P real-time PCR system (Stratagene). The oligonucleotide primer pairs (5′-3′ orientation) used for the NO production-related targets are as follows:

*CyclophilinA*-F: CGAGCTCTGAGCACTGGAGA, *CyclophilinA*-R: TGGCGTGTAAAGTCACCACC, *Nos3*-F: CGCAAGAGGAAGGAGTCTAGCA, *Nos3*-R: TCGAGCAAAGGCACAGAAGTGG.

### 2.12 Detection of nitric oxide concentration

To evaluate the changes in intracellular NO concentrations, an NO-sensitive fluorescence indicator, DAF-FM DA Assay Kit (S0020S, Beyotime), was used to label LECs. As previously described, confluent 35-mm glass-bottom microwell dishes containing cells were first washed 3 times in PBS and labeled with 2.5 μM DAF-FM DA diluted in assay buffer in the kit at 37 °C and 5% CO_2_ for approximately 20 min. Protected from the light, the dishes were washed 3 times with PBS and then phenol red-free DMEM was added. Next, the cells were visualized and imaged to observe NO production using an Olympus IX81 fluorescence microscope (Olympus Optical, Japan).

### 2.13 Power analysis

Sample sizes for most key experiments were determined by a priori power calculations using G*Power. 1) For assessing joint lymphatic drainage between RT and 4 °C groups in DMM-operated mice, we powered the experiment to detect a difference in fluorescence intensity for a two-sample t-test with an effect size of Cohen's d = 2.19 from our pilot data. Assuming a two-sided α level of 0.05 and 80% power, the required sample size was n ≥ 5 per group. 2) For assessing the effect of LIPUS in cold-stimulated DMM mice, the required sample size n ≥ 4 per group was calculated given an effect size of Cohen's d = 2.83 for a two-sided independent-samples t-test estimated from data reported in a previous study with α = 0.05 and 80% power [35]. 3) For the experiments assessing the effect of TRPV4 inhibitor HC-067047 and lymphatics ligation, a retrospective post hoc power analysis was performed for a one-way ANOVA. Based on the observed effect size (Cohen's *f* = 1.135), with an α level of 0.05, a total sample size of 18 animals (n = 6 per group), the achieved statistical power was 0.979.

### 2.14 Statistical analysis

Statistical analysis and graphing were performed using GraphPad Prism 9.0.0. Data are presented as mean ± standard error of the mean (mean ± SEM). Comparisons between two groups were performed using an unpaired Student's t-test. For comparisons among multiple groups involving a single factor, one-way analysis of variance (ANOVA) followed by Dunnett's post hoc test was used. For comparisons among multiple groups involving two factors, two-way ANOVA followed by Tukey's multiple-comparisons test was used. A p value < 0.05 was considered statistically significant (ns, P > 0.05; *P < 0.05; **P < 0.01; ***P < 0.001; ****P < 0.0001).

## 3 Results

### 3.1 Acute cold stimulation aggravates joint pain, effusion and macrophage accumulation

Destabilization of the medial meniscus (DMM) surgery was performed in 12-week-old C57BL/6J mice to establish OA models. At four weeks post-DMM surgery, a stage characterized by moderate synovitis and mild pain [22], mice were subjected to cold stimulation (4 °C) and subsequently assessed as outlined in Fig. 1A. Given that joint pain is a primary symptom exacerbated by cold exposure in OA patients, we conducted a battery of pain-related behavioral tests, including gait analysis, open field test, and weight-bearing asymmetry test. Compared with the room temperature (RT) group, the 4 °C group exhibited a significantly greater reduction in maximum contact area, swing speed, and duty cycle in the surgical hindlimb relative to the contralateral limb (Fig. 1B and C), suggesting that low temperature aggravated joint pain in DMM-operated mice. Open field tests further revealed reduced locomotor activity in DMM-operated mice under cold exposure, as evidenced by significantly decreased total distance traveled in the 4 °C group compared with the RT group during the 5-min test (Fig. 1D and E). To further evaluate mechanical pain-related behaviors, weight-bearing asymmetry and von Frey tests were assessed and the results indicated increased weight-bearing asymmetry and lower paw withdrawal thresholds in the 4 °C group (Fig. 1F and G). Collectively, these findings demonstrate that acute cold stimulation exacerbates pain in the OA joint. Additionally, similar assessments were conducted in sham-operated mice following cold stimulation, which revealed no significant worsening of pain-related behaviors (Fig. S1A–C). These results suggest that acute cold stimulation does not directly induce pain in healthy joints.

**Fig. 1:**
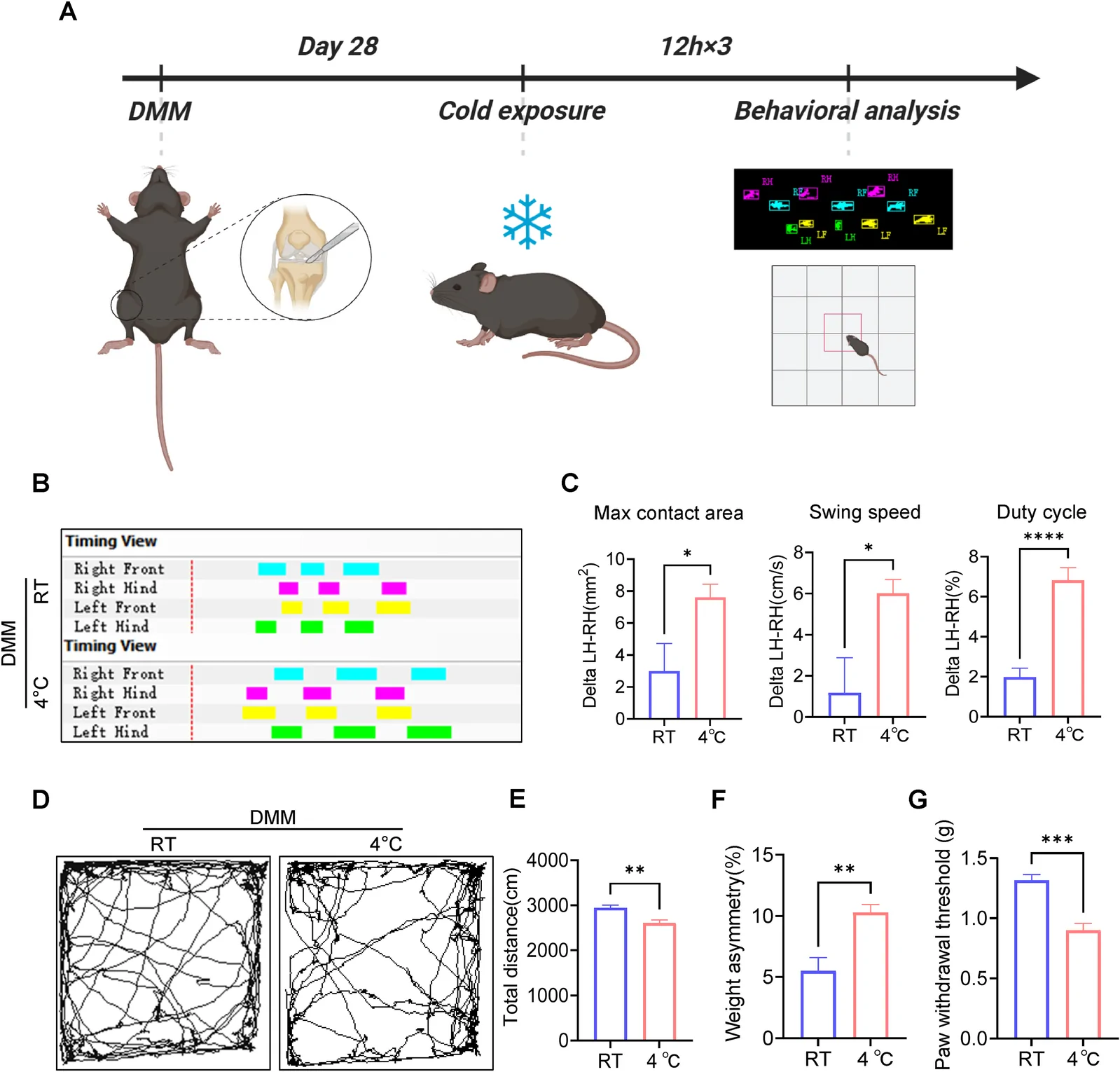
**Acute cold stimulation aggravates joint pain in DMM mouse model**. (A) Schematic illustration of the experimental design, including DMM surgery in 3-month-old male C57BL/6J mice, acute cold stimulation, and subsequent functional assessments. (B) Representative gait patterns (footprints) of DMM-operated mice in the RT and 4 °C groups visualized by the CatWalk system. (C) Quantitative analysis of gait data in the RT and 4 °C groups (n = 6). (D) Representative locomotor trajectories of mice in the RT and 4 °C groups during the open field test. (E) Quantitative analysis of the total distance traveled by each group (n = 6) in the open field test. (F) Quantitative analysis of weight bearing asymmetry in each group mice (n = 6), calculated by (weight ^LH^ - weight ^RH^)/(weight ^LH^ + weight ^RH^). (G) Quantitative analysis of von Frey paw withdrawal threshold in each group (n = 6). LH, left hind; RH, right hind. Data are presented as mean ± SEM. Statistical analysis was performed using Student's t-test, with *P < 0.05, **P < 0.01, ***P < 0.001, ****P < 0.0001.

Acute OA pain is often compounded by inflammatory pain due to synovitis and joint effusion [12], [36], which closely correlated with cold-induced pain in OA patients [37]. Therefore, we performed T2-weighted MRI to visualize joint effusion (Fig. 2A and B). The results showed that in DMM-operated mice, the joint effusion volume was significantly greater in the 4 °C group than in the RT group, whereas in sham-operated mice, no significant difference was detected between the two temperature groups (Fig. 2C and D), suggesting that acute cold stimulation exacerbates joint effusion in OA joint. Subsequently, knee joints of these mice were further analyzed for histological changes (Fig. 3A). The results showed that in sham-operated mice, there was no significant difference in OARSI scores (Fig. S2A and S2B), synovitis scores and macrophage accumulation between the two temperature groups (Fig. 3B and 3C), suggesting that acute cold stimulation did not initiate pathological changes in healthy joints. However, in DMM-operated mice, OARSI scores (Fig. S2A and S2B) and synovitis scores (Fig. 3B and D) of the 4 °C group exhibited an increasing trend compared with the RT group, although this difference did not reach statistical significance. Furthermore, immunofluorescence staining revealed that the number of synovial F4/80-positive cells in DMM-operated mice was significantly higher in the 4 °C group than in the RT group, which suggests that cold stimulation results in greater macrophage accumulation in OA joints (Fig. 3B and D). Collectively, the above findings indicate that acute cold stimulation aggravates pain, effusion and macrophage accumulation on OA joint while having negligible effects on healthy joints.

**Fig. 2:**
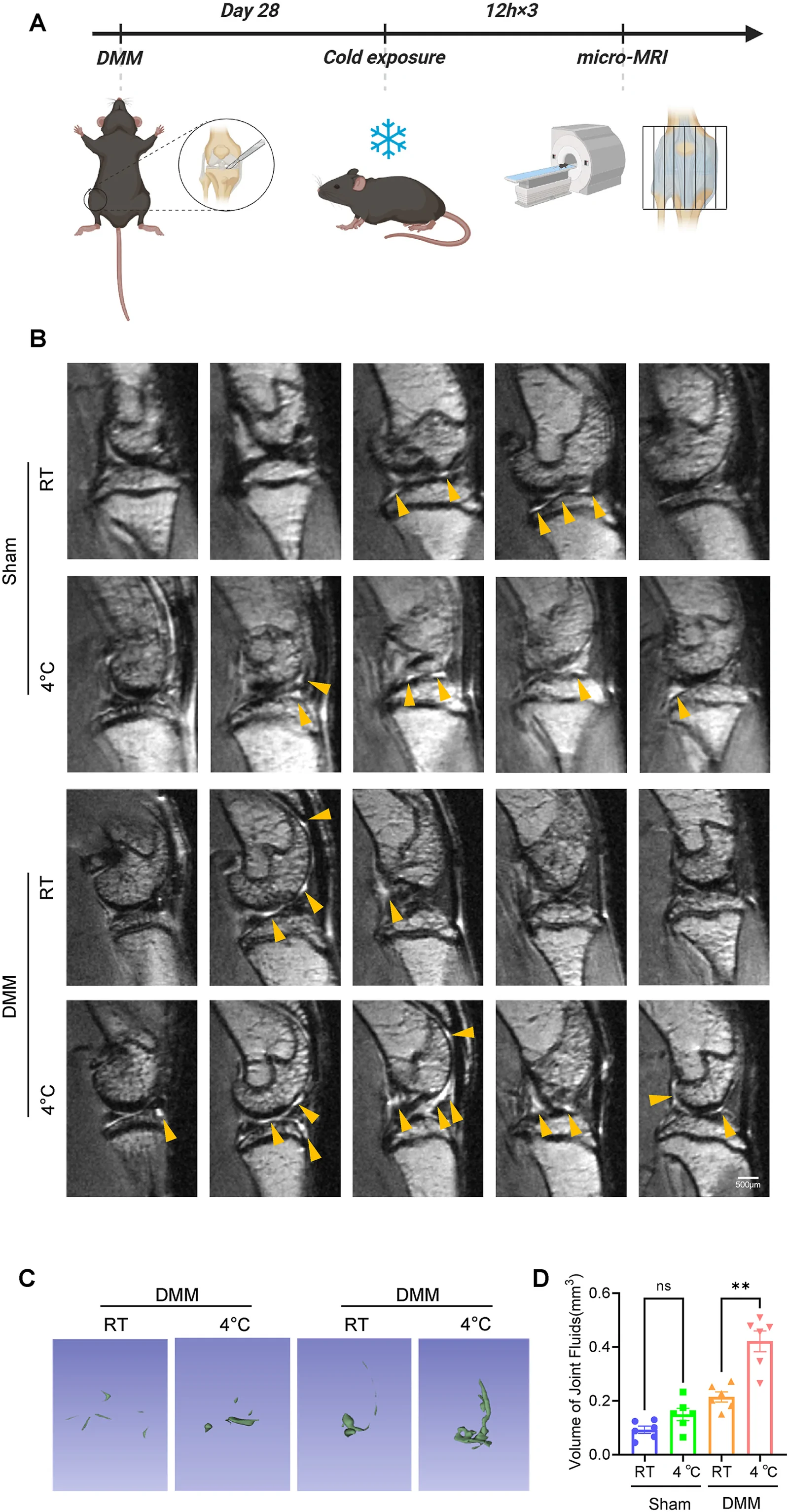
**Acute cold stimulation aggravates joint effusion in DMM mouse model.** (A) Schematic illustration of MRI scanning procedures following cold stimulation. (B) Representative T2-weighted MRI images of knee joints from Sham and DMM-operated mice in the RT and 4 °C groups. Hyperintense regions (indicated by yellow triangles) correspond to joint effusion. Scale bar: 500 μm. (C) Representative three-dimensional reconstructions of joint effusion in each group generated using 3D Slicer software. (D) Quantitative analysis of joint effusion volume in each group (n = 6). Data are presented as mean ± SEM. Statistical significance was determined using two-way ANOVA followed by Tukey's multiple comparison test (*P < 0.05, **P < 0.01).

**Fig. 3:**
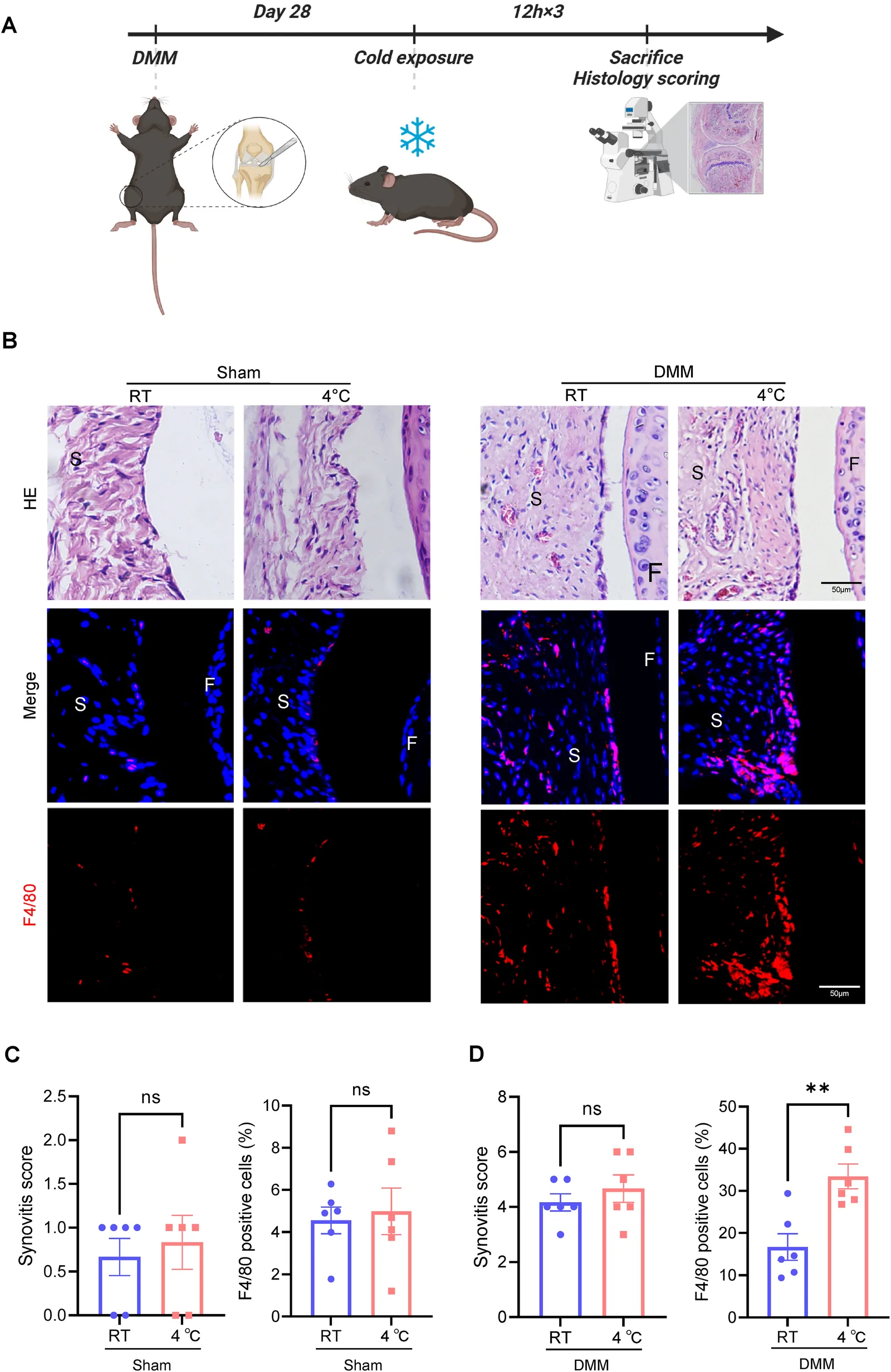
**Acute cold stimulation increased synovial macrophage accumulation in DMM mice.** (A) Schematic illustration of the timeline for histological assessment following cold stimulation. (B) Representative hematoxylin and eosin (H&E) and F4/80 immunofluorescence staining images of knee joint sections from Sham and DMM-operated mice in the RT and 4 °C groups. Scale bar: 50 μm. (C) Quantitative analysis of synovitis scores and F4/80 positive cells (%) from Sham-operated mice in the RT and 4 °C groups (n = 6). (D) Quantitative analysis of synovitis scores and F4/80 positive cells (%) from DMM-operated mice in the RT and 4 °C groups (n = 6). Data are presented as mean ± SEM. Statistical significance was determined using Student's t-test (ns, not significant, P > 0.05; **P < 0.01).

### 3.2 Acute cold stimulation impairs lymphatic drainage in OA knee joints

Given that SLS plays a crucial role in fluid drainage and inflammatory mediator clearance in the knee joint [38], and that the accumulation of macrophages in synovium is controlled by the SLS [39], we hypothesized that impaired synovial lymphatic drainage contributes to the cold-induced exacerbation of pain, effusion and macrophage accumulation in OA joints. Consistent with this hypothesis, a previous study reported that lower temperatures disrupted lymphatic contraction rhythm and reduced pump flow in rat lymphatic vessels [40]. To assess the effect of acute cold stimulation on synovial lymphatic drainage, we employed NIR-ICG imaging as illustrated in Fig. 4A. The results showed that joint clearance in DMM-operated mice was significantly lower in the 4 °C group than in the RT group, reflecting impaired synovial lymphatic drainage under acute cold stimulation (Fig. 4B and C). In addition, we analyzed the sections of knee joints and ILNs to further assess synovial lymphatic function. This approach is based on the physiological principle that intra-articular substances are absorbed by the synovium and transported via lymphatic vessels to the ILNs, enabling the evaluation of synovial influx and DLN capacity [33]. Fluorescence imaging revealed that in DMM-operated mice, there was substantial retention of FITC-dextran near the synovial lining layer in the 4 °C group, whereas in the RT group, most of the tracer was extensively diffused into the sublining layer (Fig. 4D). Quantitative analysis further revealed that the synovial influx of FITC-dextran in DMM-operated mice was significantly reduced by cold stimulation (Fig. 4E). Similarly, fluorescence intensity in the ILNs was also markedly lowered in the 4 °C group compared with the RT group (Fig. 4F and G), indicating compromised DLN capacity. Additionally, similar evaluations were conducted in Sham-operated mice. The results showed that both synovial influx and DLN capacity were decreased in the 4 °C group compared with the RT group, although the extent of this decrease was not as great as that in the DMM-operated mice (Fig. S3A–D). These findings suggest that lymphatic drainage is impaired under cold exposure in both healthy and OA joints. Collectively, the above evidence supports that impaired lymphatic drainage could serve as one of the important contributing mechanisms underlying cold-induced exacerbation of OA symptoms.

**Fig. 4:**
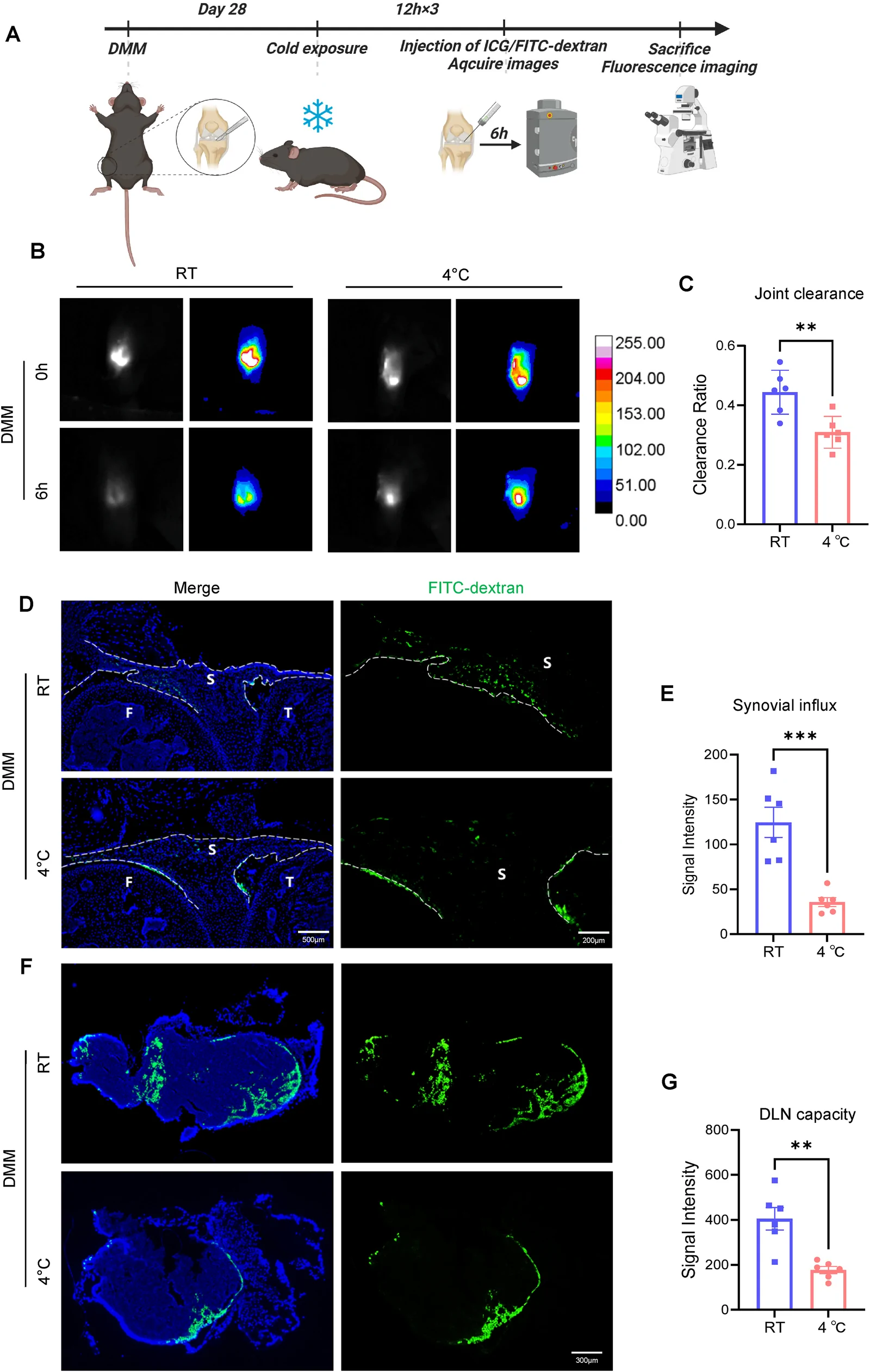
**Acute cold stimulation impairs synovial lymphatic drainage in DMM mice.** (A) Schematic illustration of lymphatic drainage function assessment following cold stimulation. (B) Representative near-infrared indocyanine green (NIR-ICG) lymphatic imaging of knee joints from DMM-operated mice in the RT and 4 °C groups at 0 and 6 h after intra-articular ICG injection. (C) Quantitative analysis of the joint clearance ratio in each group (n = 6). (D) Representative darkfield images of frozen knee sections showing synovial uptake of FITC-dextran (250 kDa) following intra-articular injection in RT and 4 °C mice. (E) Quantitative analysis of synovial dextran influx in each group (n = 6). (F) Representative darkfield images of ILN sections showing dextran accumulation following intra-articular injection in RT and 4 °C mice. Scale bar: 300 μm. (G) Quantitative analysis of DLN capacity in the RT and 4 °C groups (n = 6). Data are presented as mean ± SEM. Statistical significance was determined using Student's t-test, with *P < 0.05, **P < 0.01, ***P < 0.001.

**Fig. 5:**
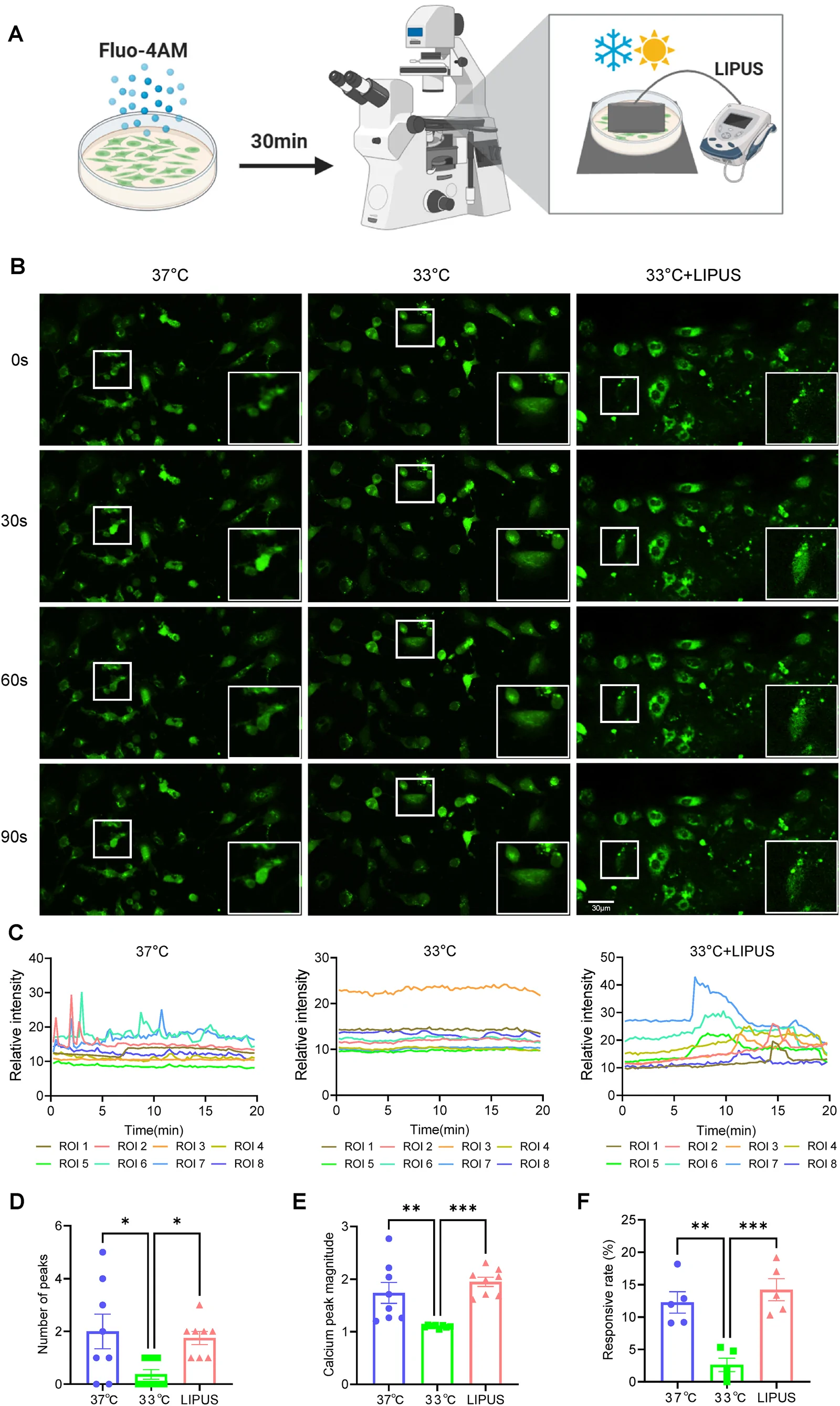
**LIPUS restores low temperature-induced suppression of calcium oscillations in LECs.** (A) Schematic illustration of the real-time calcium imaging setup involving LIPUS treatment under different temperatures. (B) Representative real-time fluorescence images showing calcium signaling in LECs within the same field of view. White rectangles indicate the same cell. Scale bar: 30 μm. (C) Representative time-course curve of relative calcium fluorescence intensity in LECs under different conditions. (D–F) Quantitative analysis of the number of calcium peaks (D, n = 8), peak magnitude (E, n = 8), and cell response rate (F, n = 5). Data are presented as mean ± SEM. Statistical significance was determined using two-way ANOVA followed by Tukey's multiple-comparisons test, with *P < 0.05, **P < 0.01, ***P < 0.001.

### 3.3 LIPUS rescues cold-suppressed calcium oscillations in LECs

Synovial lymphatic drainage is primarily driven by the spontaneous contraction of lymphatic vessels, a process tightly regulated by calcium signaling [9]. During a contraction cycle, pacemaker-generated spontaneous action potentials in LMCs trigger the opening of voltage-dependent Ca2+ channels, allowing extracellular Ca^2+^ influx and subsequent contraction via downstream myosin light chain kinase (MLCK) activation [41]. At the end of the contraction phase, high fluid shear stress activates calcium signaling in LECs, promoting nitric oxide (NO) release and subsequent LMC relaxation. This relaxation is critical for enabling stronger stretch-induced contractions in the next cycle [42]. Collectively, lymphatic pumping, aided by lymphatic valves, requires adequate diastolic relaxation for efficient lymph transport and the activation of calcium signaling in LECs is essential for continuous lymphatic pumping. In addition, previous studies demonstrated that low temperature inhibits calcium oscillations in multiple cells [43], [44], and meanwhile LIPUS attenuates OA pathogenesis through activation of calcium oscillations [18], [35]. Thus, we deduced that low temperature may dampen calcium oscillations in LECs which could be restored by LIPUS treatment.

To verify this hypothesis, we established a customized setup for live-cell calcium imaging. Briefly, LIPUS was applied directly to LECs, and live-cell images were acquired using an inverted fluorescence microscope equipped with a temperature-controlled chamber (Fig. 5A). To simulate low-temperature conditions, LECs were maintained at 33 °C. This setting was based on prior evidence that the mouse intra-articular temperature decreased and was maintained ∼33 °C when ambient temperature dropped to 4 °C [2]. As shown in Fig. 5B and Movie S1–S3, Fluo-4-loaded LECs exhibited fluorescence fluctuations indicative of active calcium oscillations in the 37 °C group. However, these active calcium signals were largely suppressed in the 33 °C group, which were restored under LIPUS treatment (Fig. 5C). Quantitative analysis further showed that over a 20-min observation period, the 37 °C group displayed robust calcium oscillations, characterized by peak frequency of 2.000 ± 0.541, peak magnitude of 1.739 ± 0.20-fold over baseline, and responsive cell proportion of 12.25 ± 1.66%. In contrast, calcium oscillations in the 33 °C group were significantly suppressed, as evidenced by reduced peak frequency (0.375 ± 0.18), attenuated peak magnitude (1.101 ± 0.01-fold over baseline), and decreased responsive cell proportion (2.63 ± 1.04%). Notably, LIPUS treatment effectively reversed this cold-induced inhibition, as illustrated by increasing peak frequency (1.750 ± 0.25), peak magnitude (1.951 ± 0.09-fold over baseline), and responsive cell proportion (14.22 ± 1.70%) (Fig. 5D–F). These findings support that LIPUS rescues LEC calcium oscillations from cold-induced suppression in vitro.

### 3.4 LIPUS improves lymphatic drainage and alleviates cold-stimulated acute exacerbation of OA phenotypes in mice

Subsequently, we applied LIPUS to DMM-operated mice after cold stimulation to evaluate its efficacy in restoring synovial lymphatic drainage in vivo. Quantitative analysis of NIR-ICG imaging revealed that LIPUS treatment effectively promoted joint clearance in DMM-operated mice subjected to cold stimulation (Fig. 6A and B). Notably, LIPUS treatment also increased joint clearance in the DMM-operated mice maintained at RT (Fig. S4A and S4B). Additionally, the FITC-dextran drainage assay showed significant increases in synovial influx and DLN capacity following LIPUS treatment in the DMM-operated mice that had undergone cold stimulation (Fig. 6C–F). These lymphatic-promoting effects were also observed in mice maintained at RT (Fig. S4C and S4D). The above findings suggest that LIPUS is capable of promoting lymphatic drainage under both normal and cold stimulation conditions.

**Fig. 6:**
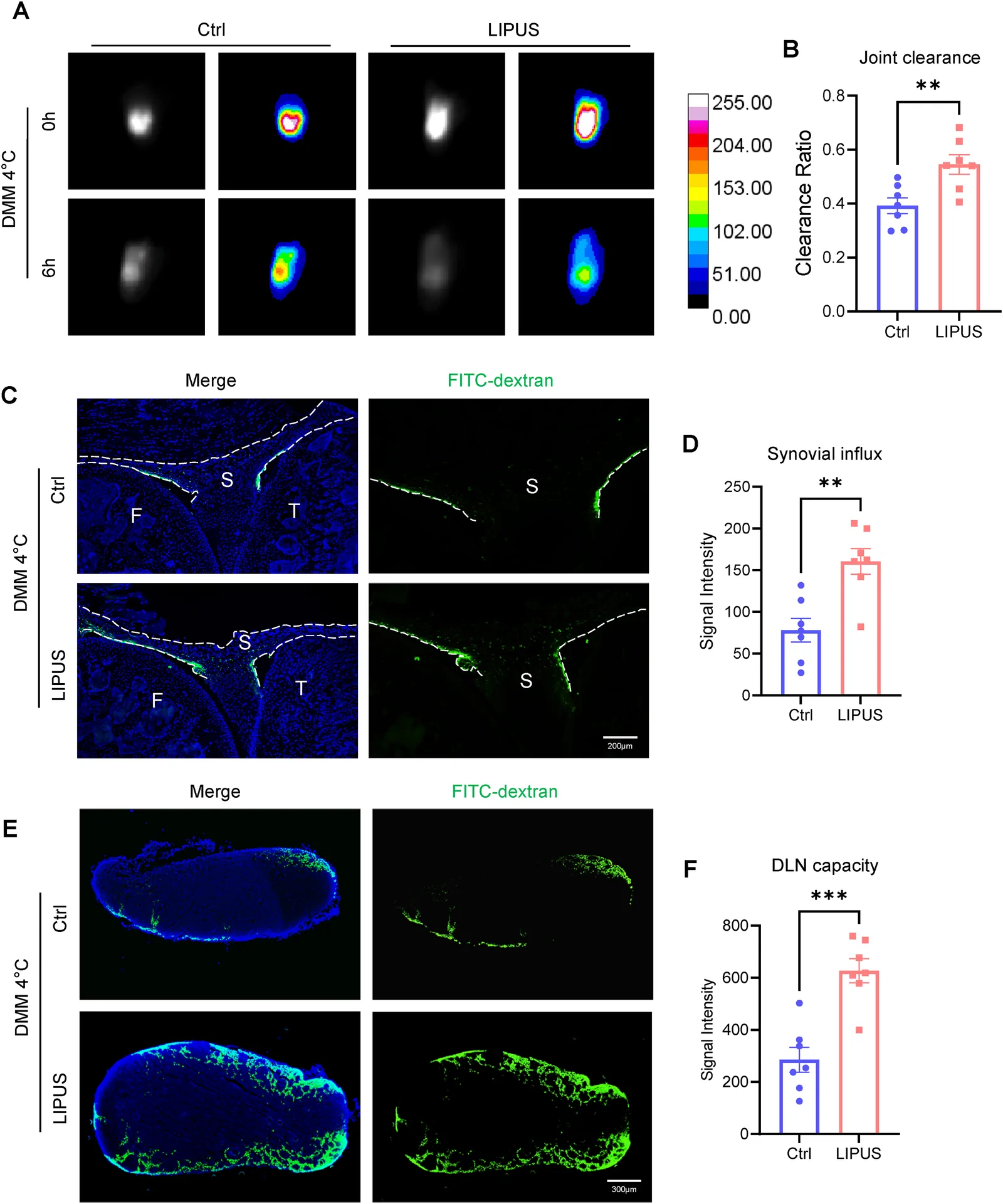
**LIPUS ameliorates cold-induced lymphatic drainage dysfunction in DMM joints.** (A) Representative NIR-ICG images of knee joints from DMM 4 °C mice in the control (Ctrl) and LIPUS groups at 0 and 6 h following intra-articular ICG injection. (B) Quantitative analysis of the joint clearance ratio in the control and LIPUS groups (n = 7). (C) Representative darkfield images of frozen knee sections showing synovial uptake of FITC-dextran (250 kDa) following intra-articular injection in the control and LIPUS groups. (D) Quantitative analysis of synovial dextran influx in each group (n = 7). (E) Representative darkfield images of ILN sections showing dextran accumulation following intra-articular injection in the control and LIPUS groups. (F) Quantitative analysis of draining lymph node (DLN) capacity in each group (n = 7). Data are presented as mean ± SEM. Statistical significance was determined using Student's t-test (**P < 0.01, ***P < 0.001).

To determine whether improved drainage contributes to attenuated OA phenotypes worsened by acute cold stimulation, the therapeutic effects of LIPUS were assessed on DMM-operated mice subjected to cold stimulation with or without LIPUS treatment. The results showed that joint effusion was significantly reduced in the LIPUS treatment group compared with the control group (Fig. 7A and B). Meanwhile, synovitis scores of the LIPUS treatment group exhibited a decreasing trend compared with the control group, although this difference did not reach statistical significance (Fig. 7C and D). However, there were significantly fewer F4/80 positive cells located in the synovial sublining layer in the LIPUS treatment group compared with the control group (Fig. 7C and E), indicating attenuated synovial macrophage accumulation following LIPUS treatment. In addition, LIPUS treatment did not significantly affect cartilage matrix degradation in the short term (Fig. S5A and S5B). Furthermore, gait analysis revealed that, compared with the control group, LIPUS treatment significantly reduced asymmetry in swing speed, maximum contact area, and duty cycle between the DMM-operated and contralateral hindlimbs (Fig. 7F), indicating ameliorated gait abnormalities. Furthermore, LIPUS treatment also increased the paw withdrawal threshold and lowered weight-bearing asymmetry (Fig. 7G and H), suggesting an alleviation of pain in cold-stimulated OA joints. Interestingly, LIPUS treatment appeared to attenuate OA phenotypes in DMM-operated mice maintained at RT; However, most of these differences did not reach statistical significance, except for joint effusion volume (Fig. S6A–G), possibly because the relatively short experimental duration limited our ability to detect structural or pathological changes. Collectively, these findings illustrated that LIPUS could improve lymphatic drainage and attenuate cold-stimulated exacerbation of joint pain, effusion and macrophage accumulation in OA joints.

**Fig. 7:**
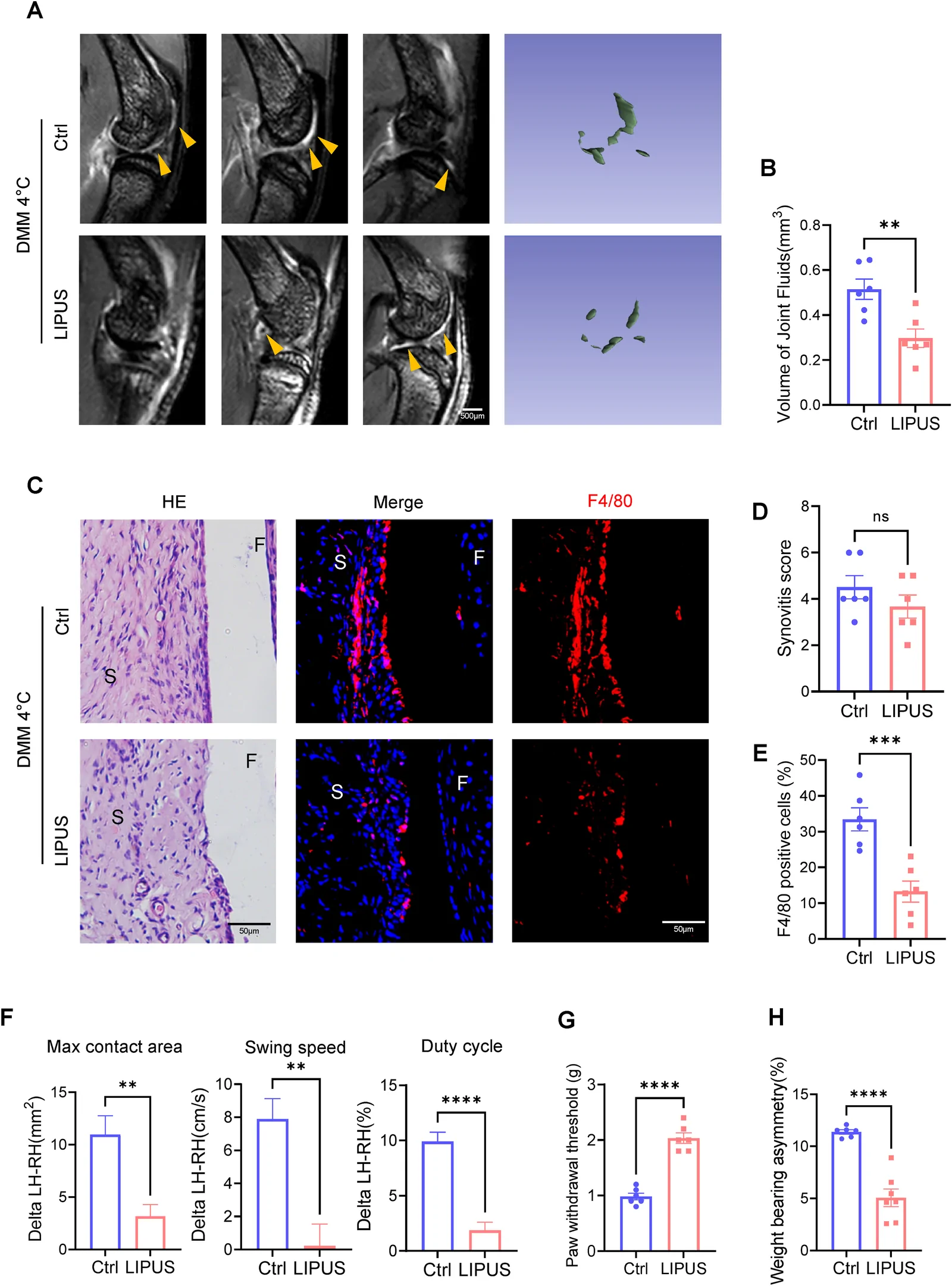
**LIPUS attenuates joint pain, effusion and macrophage accumulation in cold-stimulated DMM mice.** (A) Representative T2-weighted MRI images of knee joints and three-dimensional reconstructions of joint effusion from DMM 4 °C mice in the control (Ctrl) and LIPUS groups. Hyperintense regions (indicated by yellow triangles) correspond to joint effusion. Scale bar: 500 μm. (B) Quantitative analysis of joint effusion volume in each group (n = 6). (C) Representative HE staining and F4/80 immunofluorescent staining images of knee joint sections from DMM 4 °C mice in the control and LIPUS groups. Scale bar: 50 μm. (D) Quantitative analysis of synovitis scores in each group (n = 6). (E) Quantitative analysis of F4/80 positive cells (%) in each group (n = 6). (F) Quantitative analysis of gait data using the CatWalk system in each group (n = 6). (G) Quantitative analysis of weight-bearing asymmetry in each group (n = 6). (H) Quantitative analysis of von Frey paw withdrawal threshold in each group (Ctrl, n = 6; LIPUS, n = 7). Data are presented as mean ± SEM. Statistical analysis was performed using Student's t-test, with ns, P > 0.05; **P < 0.01, ***P < 0.001, ****P < 0.0001.

### 3.5 LIPUS promotes calcium oscillations and lymphatic drainage through activation of TRPV4 in LECs

As a non-invasive physical therapy modality, LIPUS exerts its biological effects primarily through the transduction of mechanical forces into biochemical signals. Within this complex mechanotransduction network, TRPV4 has been identified as a key mechanosensitive calcium channel, playing a central role in mediating LIPUS-induced intracellular calcium oscillations and downstream physiological responses [18], [45]. Furthermore, TRPV4 also acts as a temperature-sensitive ion channel with an activation range of 27 °C-42 °C [46], and its activation increases with rising temperature [47]. In addition, TRPV4 plays a dominant role in the temperature-dependent regulation of lymphatic flow [14], and it is predominantly expressed in LECs rather than LMCs [48]. Therefore, we deduced that LIPUS may activate calcium oscillations in LECs via TRPV4. Subsequently, HC-067047 was used to selectively inhibit TRPV4 [49]. Living cell imaging showed that LIPUS-induced calcium oscillations were significantly inhibited in SVEC4-10 cells pretreated with HC-067047 compared with those pretreated with saline (Fig. 8A–E and Movie S4, S5). These findings indicate that TRPV4 contributes to LIPUS-induced calcium oscillations in LECs.

**Fig. 8:**
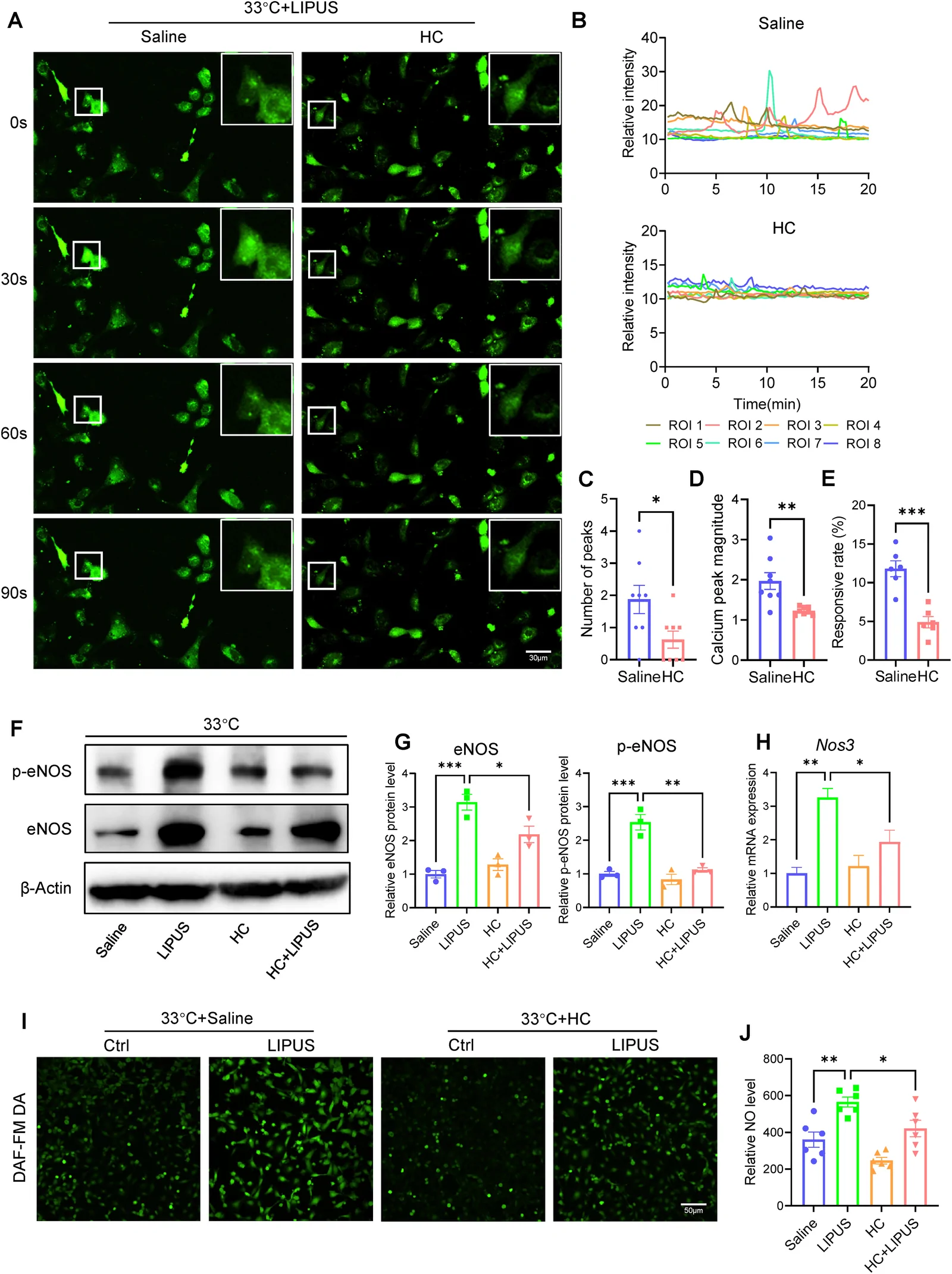
**TRPV4 blockade inhibits LIPUS-induced calcium oscillations and NO production in vitro.** (A) Representative real-time fluorescence images showing calcium signaling in LECs pretreated by saline and HC-067047 within the same field of view. White rectangles indicate the same cell. Scale bar: 30 μm. (B) Representative time-course curve of relative calcium fluorescence intensity in LECs under different conditions. (C–E) Quantitative analysis of the number of calcium peaks (C, n = 8), peak magnitude (D, n = 8), and cell response rate (E, n = 6). (F) Western blot detection of eNOS and p-eNOS expression in LECs under different treatment conditions. (G) Quantitative analysis of protein expression levels of eNOS and p-eNOS in LECs under different treatment conditions (n = 3). (H) Quantitative analysis of mRNA expression levels of *Nos3* in LECs under different treatment conditions (n = 3). (I) Representative fluorescent images of LECs incubated with DAF-FM DA from different groups. (J) Quantitative analysis of relative NO level in different groups (n = 6). Data are presented as mean ± SEM. Statistical significance was determined using two-way ANOVA followed by Tukey's multiple-comparisons test, with *P < 0.05; **P < 0.01, ***P < 0.001.

NO-mediated lymphatic relaxation, together with lymphatic contraction, constitutes an essential physiological basis for maintaining lymphatic pumping [42]. Under physiological conditions, NO is predominantly generated by eNOS [50], whose phosphorylated activation is Ca^2+^-dependent. Interestingly, we found that p-eNOS was significantly decreased in SVEC4-10 cells cultured at 33 °C, which has no significant influence on total eNOS (Fig. S7A and S7B). Moreover, the production of NO in SVEC4-10 cells was also decreased at 33 °C (Fig. S7C and S7D). Furthermore, LIPUS treatment significantly increased eNOS and p-eNOS levels in SVEC4-10 cells maintained at 33 °C, whereas these effects were markedly suppressed by HC-067047 (Fig. 8F–H). Consistently, the production of NO was raised by LIPUS treatment in SVEC4-10 cells maintained at 33 °C, and TRPV4 inhibition reversed this increase (Fig. 8I and J). Taken together, the above findings suggest that LIPUS induces intracellular calcium oscillations in LECs, thereby promoting eNOS phosphorylation and activation and consequently increasing NO production. TRPV4 acts as a key upstream mediator of this signaling pathway.

Subsequently, we investigated the role of TRPV4 in LIPUS-induced lymphatic drainage in DMM-operated mice under cold exposure. Compared with the saline-treated group, mice receiving intra-articular administration of HC-067047 exhibited significantly reduced joint clearance, which was consistent with the results observed in mice subjected to lymphatic vessel ligation (Fig. 9A and B). Furthermore, LIPUS-induced DLN capacity was also markedly suppressed by TRPV4 inhibition or lymphatic drainage blockade (Fig. 9C and E), indicating that LIPUS-induced lymphatic drainage was largely dependent on TRPV4. Histological evaluation experiments revealed that the synovitis scores were not significantly different among saline group, HC-067047 group and lymphatic vessel ligation group co-treated with LIPUS (Fig. 9D and F). However, the accumulation of F4/80-positive synovial macrophages was significantly increased by HC-067047 or lymphatic vessel ligation (Fig. 9D and G), and the therapeutic effects of LIPUS in alleviating gait abnormalities and joint pain were also markedly suppressed by TRPV4 inhibition or lymphatic drainage blockade (Fig. S8A and S8B), suggesting that LIPUS ameliorates cold-induced worsening of OA phenotypes by promoting TRPV4-mediated lymphatic drainage.

**Fig. 9:**
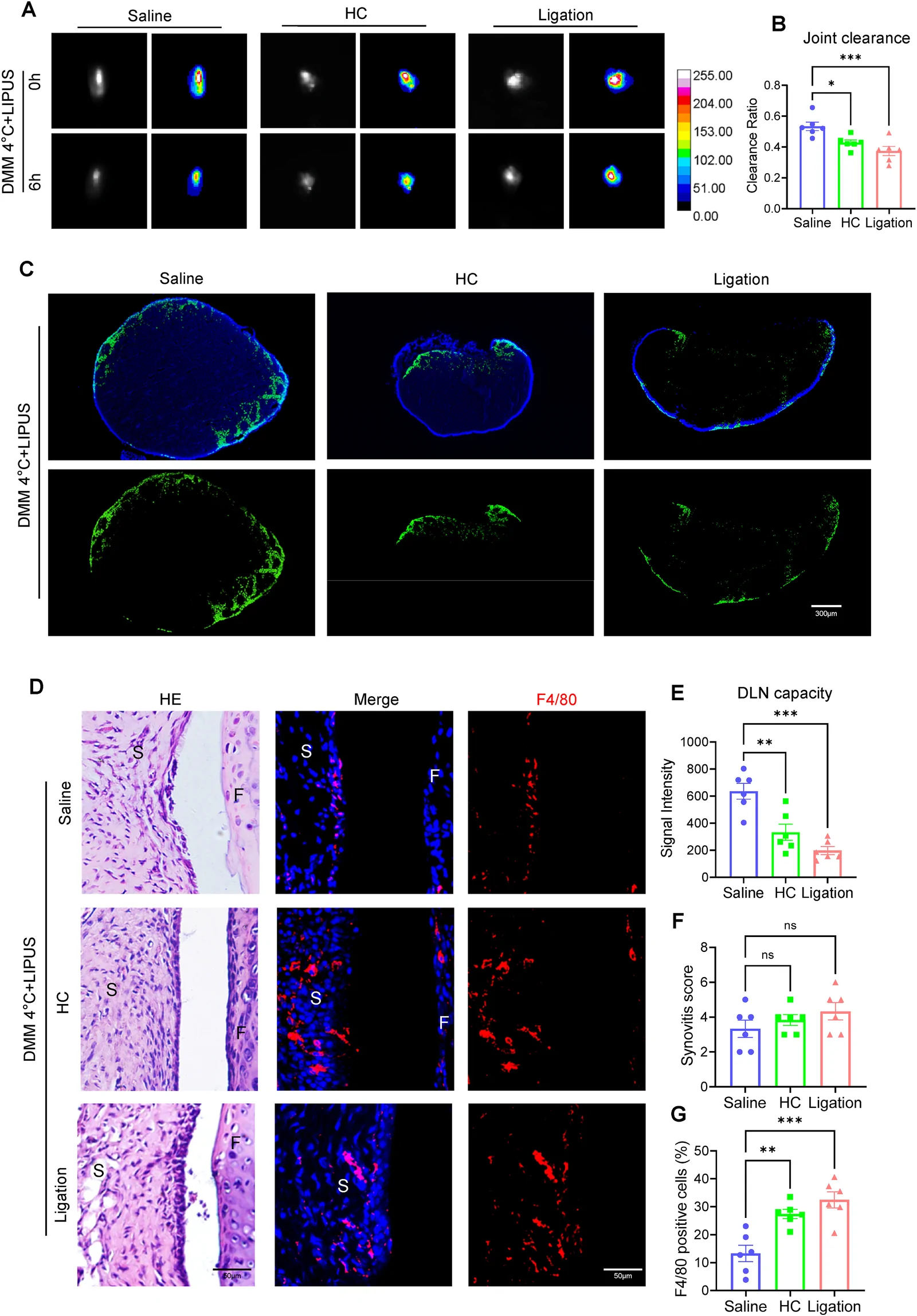
**TRPV4 blockade inhibits LIPUS-improved lymphatic drainage in vivo.** (A) Representative NIR-ICG lymphatic imaging of knee joints from DMM 4 °C + LIPUS mice in the Saline, HC and Ligation groups at 0 and 6 h after intra-articular ICG injection. (B) Quantitative analysis of the joint clearance ratio in each group (n = 6). (C) Representative darkfield images of ILN sections showing dextran accumulation following intra-articular injection in the Saline, HC and Ligation groups. Scale bar: 300 μm. (D) Representative HE staining and F4/80 immunofluorescent staining images of knee joint sections in the Saline, HC and Ligation groups. Scale bar: 50 μm. (E) Quantitative analysis of DLN capacity in each group (n = 6). (F) Quantitative analysis of synovitis scores in each group (n = 6). (G) Quantitative analysis of F4/80 positive cells (%) in each group (n = 6). Data are presented as mean ± SEM. Statistical significance was determined using one-way ANOVA followed by Dunnett's multiple-comparisons test, with *P < 0.05; **P < 0.01, ***P < 0.001.

Collectively, our findings demonstrate that LIPUS promotes lymphatic drainage by inducing TRPV4-mediated calcium oscillations in LECs and attenuates cold-stimulated exacerbation of joint pain, effusion and macrophage accumulation in OA joints (Fig. 10).

**Fig. 10:**
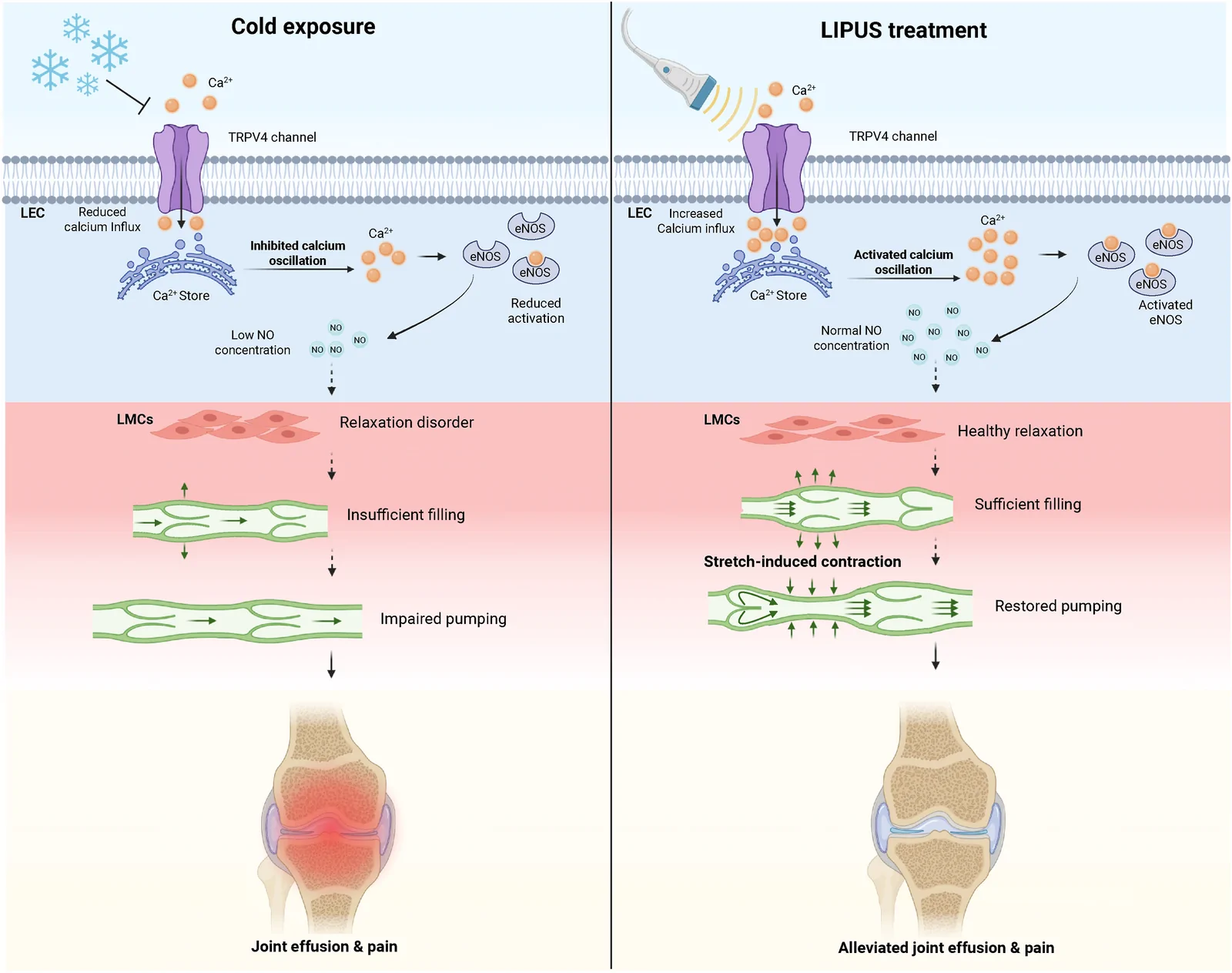
**Schematic diagram illustrates the roles and mechanisms of LIPUS on synovial lymphatic drainage and OA treatment.** LIPUS activates TRPV4-contributed calcium oscillations in LECs, subsequently increasing intracellular Ca^2+^ levels. This promotes eNOS phosphorylation and activation, restoring NO production, which is otherwise inhibited under cold conditions. Restored NO levels facilitate the proper relaxation of LMCs, allowing for adequate lymphatic filling (hypothetical model). Consequently, this leads to robust stretch-mediated contraction and improves SLS drainage function. Solid arrows indicate the experimentally demonstrated steps in this study, while dashed arrows represent the downstream mechanisms supported by previous literature. The schematic diagram was created in https://BioRender.com.

## 4 Discussion

Cold exposure has substantial influence on arthritis. An animal study found that prolonged low-temperature exposure aggravates cartilage degeneration and promotes OA progression in mice [2]. A multicenter clinical study from Europe reported that approximately two-thirds of OA patients are weather-sensitive, with exacerbated joint pain in cold weather [51], [52]. Clinically, cold pain threshold tests showed that OA patients are more sensitive to temperature drops, and even minor reductions can induce significant joint pain [53]. In this study, we further revealed that short-lived cold exposure impairs lymphatic drainage, which serves as an important, albeit not exclusive, contributing mechanism underlying cold-induced exacerbation of OA pain and joint effusion in mice. Furthermore, cold exposure did not induce significant pain or gait abnormalities in normal joints with impaired lymphatic drainage, suggesting that although impaired lymphatic drainage alone may be insufficient to elicit overt pain, it may act synergistically with algogenic substances, such as higher levels of pro-inflammatory mediators [54], to exacerbate symptoms in an already diseased OA joint. Collectively, the LIPUS treatment is effective in promoting lymphatic drainage and alleviating joint pain, effusion and inflammatory cell accumulation in OA joints.

Synovial fluid is derived from capillary filtration and synovial secretion. Capillaries provide the plasma filtrate containing water, electrolytes, while synovial cells produce hyaluronic acid and lubricin [55]. Capillary filtration is governed by Starling forces, determined by the pressure difference across capillary walls. The homeostasis of synovial fluid is controlled by lymphatic drainage, clearing excess fluid and macromolecules. There exists a small amount of synovial fluid in the joint cavity under physiological conditions, and its volume typically increases during active joint inflammation [56]. Our data found that under acute cold stimulation, the synovial lymphatic drainage was impaired which promoted effusion in OA joints. However, short-lived cold exposure was also thought to increase knee joint blood flow in arthritis models [57], indicating that the increased joint effusion in our study might involve not only impaired lymphatic drainage but also potentially increased synovial fluid production due to elevated knee blood flow under cold stimulation.

Spontaneous contraction is the fundamental mechanism driving lymphatic drainage [9], in which eNOS serves as a key enzyme for NO production and maintenance of normal lymphatic relaxation-contraction rhythm [42]. Conversely, disruption of this relaxation-contraction rhythm impairs lymph flow [58]. Mechanistically, LEC-generated NO diffuses to surrounding LMCs and reaches sufficient concentrations to regulate LMC relaxation by activating intracellular cGMP signaling [38], [59]. This mechanism underlies the stretch-induced contraction of lymphatic vessels; that is, improved relaxation allows greater lymphatic filling, which in turn produces stronger stretch on lymphatic vessels and subsequent contraction, thereby promoting more efficient lymph transport [42]. Furthermore, calcium signaling serves as the key signaling regulating eNOS phosphorylation and activation [60], and blocking calcium signaling significantly impairs spontaneous lymphatic contraction [61]. The calcium oscillation can be activated by multiple ion channels including Piezo1, Piezo2, and TRP family members, which typically occurs within seconds to minutes upon stimulation [62], [63]. Among them, TRPV4 is a temperature-sensitive ion channel that is highly expressed in LECs rather than LMCs [48]. Furthermore, TRPV4 plays a dominant role in the temperature modulation of intrinsic contractility and lymph flow of lymphatics through regulating calcium signaling in LECs [14], [64]. Similarly, our data show that calcium activities of LECs and their NO production decrease with temperature drops and these decreases are restored by LIPUS treatment. Notably, LIPUS-induced calcium activation exhibits a delayed response, with active calcium oscillations occurring a few minutes post-treatment rather than immediately upon stimulation, indicating that LIPUS-mediated lymphatic modulation may depend on calcium signaling cascades and involve cumulative effects [65]. Collectively, our data reveal that LIPUS could improve contractile-relaxation rhythm under cold stimulation and promote lymphatic drainage via the TRPV4/Ca^2+^/eNOS/NO signaling axis.

Lymphatic drainage dysfunction plays a crucial role in the pathogenesis of skeletal diseases. Consequently, modulating lymphatic function alters these pathological outcomes. For example, aberrant local lymphatic formation leads to trauma-induced heterotopic ossification in mice [66]. Improvement of SLS function via intraarticular VEGF-C administration reduces OA tissue damage in aged mice [33]. Manual lymphatic drainage reduces early-stage lower extremity edema and pain following total knee arthroplasty [67]. Nevertheless, there is still a lack of strategies to promote lymphatic drainage for disease therapy. As a safe and non-invasive therapeutic strategy, LIPUS has been widely utilized to promote fracture healing and wound repair [16]. Our study further demonstrates that LIPUS is efficient in promoting lymphatic drainage. Therefore, it is reasonable to consider its potential application for other skeletal diseases associated with lymphatic drainage dysfunction, such as heterotopic ossification [68] and edema caused by sports injuries [69].

This study has several limitations. OA models were established in 12-week-old mice by surgical destabilization of the medial meniscus, which represents a trauma-induced OA model that is distinct from age-related spontaneous OA. Consequently, our findings may not be directly applicable to age-related OA. Meanwhile, cold stimulation was applied systemically rather than locally. Although the core temperature of mice was maintained stable, potential systemic influences from other tissues or organs on the local joints cannot be ruled out. In addition, systemic cold exposure may aggravate pain through neural sensitization [70], [71]. Furthermore, it may also activate the sympathetic nervous system, thereby promoting vasoconstriction, increasing capillary hydrostatic pressure, and augmenting synovial fluid production [72]. Therefore, the acute cold-induced exacerbation of joint pain and effusion may not be attributable solely to impaired local synovial lymphatic drainage. Further studies employing localized cold stimulation models will help to avoid these shortcomings. Notably, this study exclusively used male mice, thereby failing to reveal sex differences in cold-induced exacerbation of OA phenotypes and the LIPUS-induced therapeutic effects. Moreover, LIPUS has been reported to alleviate OA pain through direct analgesic effects on peripheral nerves or by reducing inflammatory cytokines from macrophages [24], [73], suggesting that LIPUS may reduce pain through pathways independent of lymphatic drainage. Besides, whether our findings, based on short-term cold exposure, can be extrapolated to arthritis under prolonged cold exposure remains to be determined [74]. Nevertheless, this study provides new insights into the mechanisms underlying cold-exacerbated OA phenotypes and raises the possibility of using LIPUS as a lymphatic-promoting therapy, though its efficacy in alleviating “cold knees” in OA patients remains to be investigated.

## Ethics declaration

This study was conducted in accordance with the NIH guidelines for the Care and Use of Laboratory Animals. This study was approved by the Laboratory Animal Welfare Ethics Committee of the Army Medical University (Approval No. AMUWEC20237408).

## Consent for publication

All authors have reviewed the final version of the manuscript and approve it for publication.

## Data availability statement

The datasets supporting the conclusions of this article are included within the article and its Supplementary Materials.

## Declaration of generative AI in scientific writing

No generative artificial intelligence (AI) or AI-assisted technologies were used in the preparation of this manuscript.

## Declaration of competing interests

The authors have no conflicts of interest relevant to this article.

## Abbreviations

ApoE: Apolipoprotein E
OA: osteoarthritis
LMCs: lymphatic muscle cells
DLNs: draining lymph nodes
ILNs: iliac lymph nodes
SLS: synovial lymphatic system
Ctrl: control
LIPUS: low-intensity pulsed ultrasound
TRPV4: transient receptor potential vanilloid 4
DMM: destabilization of the medial meniscus
LECs: lymphatic endothelial cells
MMTL: medial meniscotibial ligament
MRI: magnetic resonance imaging
H&E: Hematoxylin and Eosin
OARSI: Osteoarthritis Research Society International
NIR-ICG: near-infrared indocyanine green
ROI: regions of interest
RT: room temperature
FITC: fluorescein isothiocyanate
MLCK: myosin light chain kinase
ns: not significant
NO: nitric oxide
eNOS: endothelial nitric oxide synthase
LH: left hind
RH: right hind
SEM: standard error of the mean

## Appendix A Supplementary data

Figure S1 **Influence of cold exposure on pain of sham-operated joints.** (A) Representative gait patterns (footprints) of Sham mice in the RT and 4 °C groups visualized by the CatWalk system. (B) Quantitative analysis of gait data in the RT and 4 °C groups (n = 6). (C) Quantitative analysis of von Frey paw withdrawal threshold in the RT and 4 °C groups (n = 6). Data are presented as mean ± SEM. Statistical analysis was performed using Student's t-test, with ns, P > 0.05.

Figure S2 **Influence of cold exposure on cartilage degeneration.** (A) Representative SO/FG staining images of knee joint sections from Sham and DMM-operated mice in the RT and 4 °C groups. Scale bar: 100 μm. (B) Quantitative analysis of OARSI scores from Sham and DMM-operated mice in the RT and 4 °C groups (n = 6). Data are presented as mean ± SEM. Statistical significance was determined using two-way ANOVA followed by Tukey's multiple comparison test (ns, not significant, P > 0.05; *P < 0.05).

Figure S3 **Influence of cold exposure on synovial lymphatic drainage in sham-operated mice.** (A) Representative NIR-ICG lymphatic imaging of knee joints from Sham-operated mice in the RT and 4 °C groups at 0 and 6 h after intra-articular ICG injection. (B) Quantitative analysis of the joint clearance ratio in each group (n = 6). (C) Representative darkfield images of ILN sections showing dextran accumulation following intra-articular injection in RT and 4 °C mice. (D) Quantitative analysis of DLN capacity in the RT and 4 °C groups (n = 6). Data are presented as mean ± SEM. Statistical significance was determined using Student's t-test, with *P < 0.05, **P < 0.01.

Figure S4 **The effects of LIPUS treatment on synovial lymphatic drainage in DMM-operated mice stayed at RT.** (A) Representative NIR-ICG lymphatic imaging of knee joints from DMM RT mice in the control (Ctrl) and LIPUS groups at 0 and 6 h after intra-articular ICG injection. (B) Quantitative analysis of the joint clearance ratio in the control and LIPUS groups (n = 6). (C) Representative darkfield images of ILN sections showing dextran accumulation following intra-articular injection in the Control and LIPUS groups. (D) Quantitative analysis of DLN capacity in each group (n = 7). Data are presented as mean ± SEM. Statistical significance was determined using Student's t-test, with *P < 0.05, **P < 0.01.

Figure S5 **Influence of LIPUS treatment on cartilage degeneration.** (A) Representative SO/FG staining images of knee joint sections from control (Ctrl) and LIPUS-treated mice in the DMM RT and DMM 4 °C groups. Scale bar: 100 μm. (B) Quantitative analysis of OARSI scores from control and LIPUS-treated mice in the DMM RT and DMM 4 °C groups (n = 6). Data are presented as mean ± SEM. Statistical significance was determined using two-way ANOVA followed by Tukey's multiple comparison test (ns, P > 0.05).

Figure S6 **The effects of LIPUS treatment on joint pain, effusion and macrophage accumulation in DMM-operated mice stayed at RT.** (A) Representative T2-weighted MRI images of knee joints and three-dimensional reconstructions of joint effusion from DMM RT mice in the control (Ctrl) and LIPUS groups. Hyperintense regions (indicated by yellow triangles) correspond to joint effusion. Scale bar: 500 μm. (B) Quantitative analysis of joint effusion volume in each group (n = 6). (C) Representative HE staining and F4/80 immunofluorescence staining images of knee joint sections from DMM RT mice in the Control and LIPUS groups. Scale bar: 50 μm. (D) Quantitative analysis of synovitis scores of each group (n = 6). (E) Quantitative analysis of F4/80 positive cells (%) in each group (n = 6). (F) Quantitative analysis of gait data using the CatWalk system in each group (n = 6). (G) Quantitative analysis of von Frey paw withdrawal threshold in each group (n = 6). Data are presented as mean ± SEM. Statistical analysis was performed using Student's t-test, with ns, P > 0.05; *P < 0.05.

Figure S7 **The influence of low temperature on levels of eNOS, p-eNOS and NO in LECs.** (A) Western blot detection of p-eNOS and eNOS expression in LECs in 37 °C and 33 °C groups. (B) Quantitative analysis of protein expression levels of p-eNOS in LECs under different treatment conditions (n = 3). (C) Representative fluorescent images of LECs incubated with DAF-FM DA from 37 °C to 33 °C groups. (D) Quantitative analysis of relative NO level in 37 °C and 33 °C groups (n = 6). Data are presented as mean ± SEM. Statistical analysis was performed using Student's t-test, with *P < 0.05, ***P < 0.001.

Figure S8 **The effects of TRPV4 blockade on LIPUS-induced locomotor activity improvement and joint pain alleviation.** (A) Quantitative analysis of gait data using the CatWalk system in Saline, HC and Ligation groups (n = 6). (B) Quantitative analysis of von Frey paw withdrawal threshold in Saline, HC and Ligation groups (n = 6). Data are presented as mean ± SEM. Statistical significance was determined using one-way ANOVA followed by Dunnett's multiple-comparisons test, with *P < 0.05; **P < 0.01, ***P < 0.001.
