# Supplementary material for: LIPUS promotes calcium oscillations to improve lymphatic drainage and alleviates cold-stimulated pain and effusion in osteoarthritis joints
Source: J Orthop Translat. 2026 Oct 24;61:101242. doi: 10.1016/j.jot.2026.101242 (PMC13721939; doi:10.1016/j.jot.2026.101242)
Supplement: Supplementary file 1 [file mmc1.pdf]

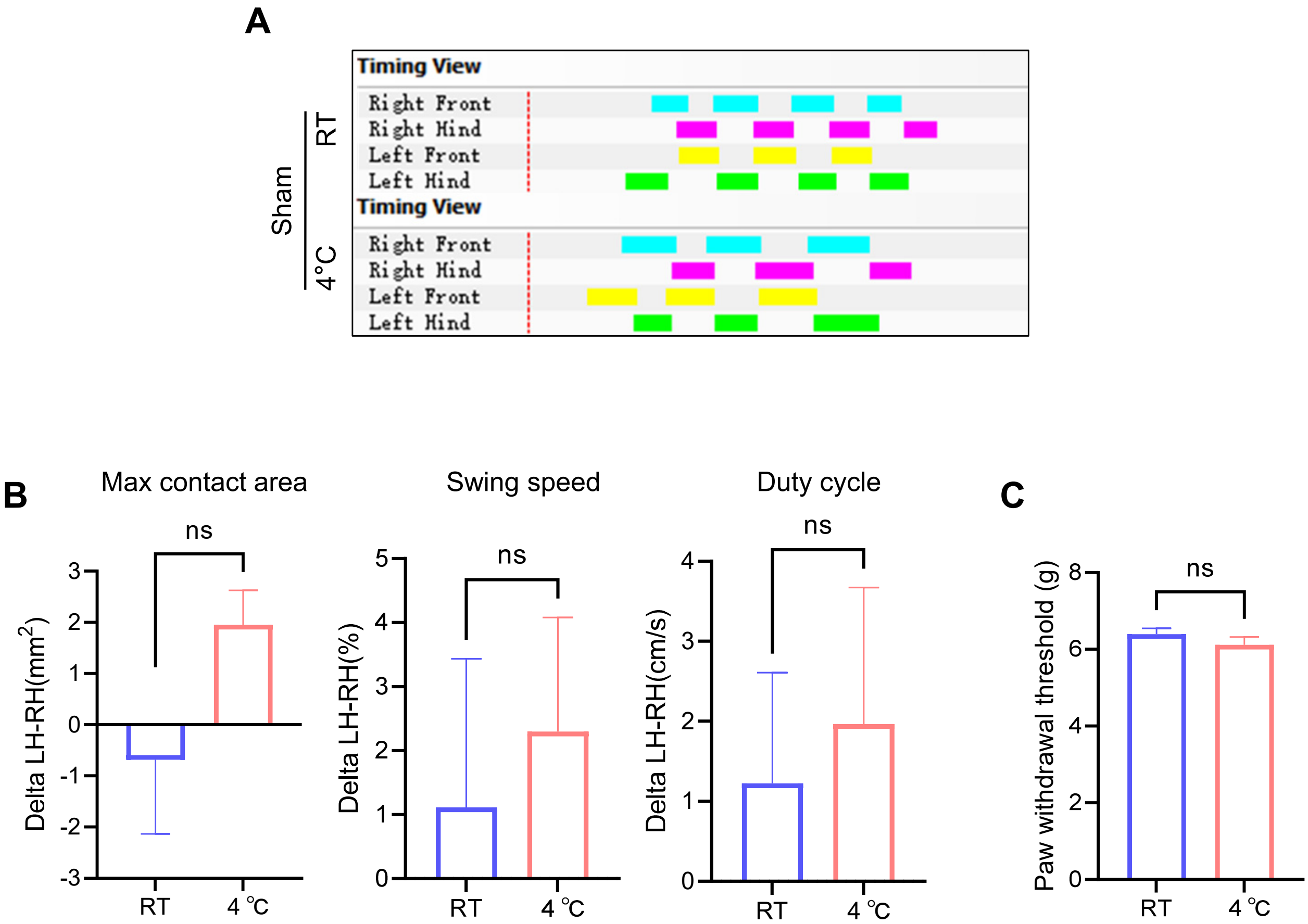
A
Timing View
Right Front
Right Hind
Left Front
Left Hind
Timing View
Right Front
Right Hind
Left Front
Left Hind
Sham
RT
4°C
B
Max contact area
Swing speed
Duty cycle
ns
Delta LH-RH(mm2)
Delta LH-RH(%)
Delta LH-RH(cm/s)
RT
4 ℃
C
Paw withdrawal threshold (g)
ns
RT
4 ℃
