# Supplementary material for: LIPUS promotes calcium oscillations to improve lymphatic drainage and alleviates cold-stimulated pain and effusion in osteoarthritis joints
Source: J Orthop Translat. 2026 Oct 24;61:101242. doi: 10.1016/j.jot.2026.101242 (PMC13721939; doi:10.1016/j.jot.2026.101242)
Supplement: Supplementary file 2 [file mmc2.pdf]

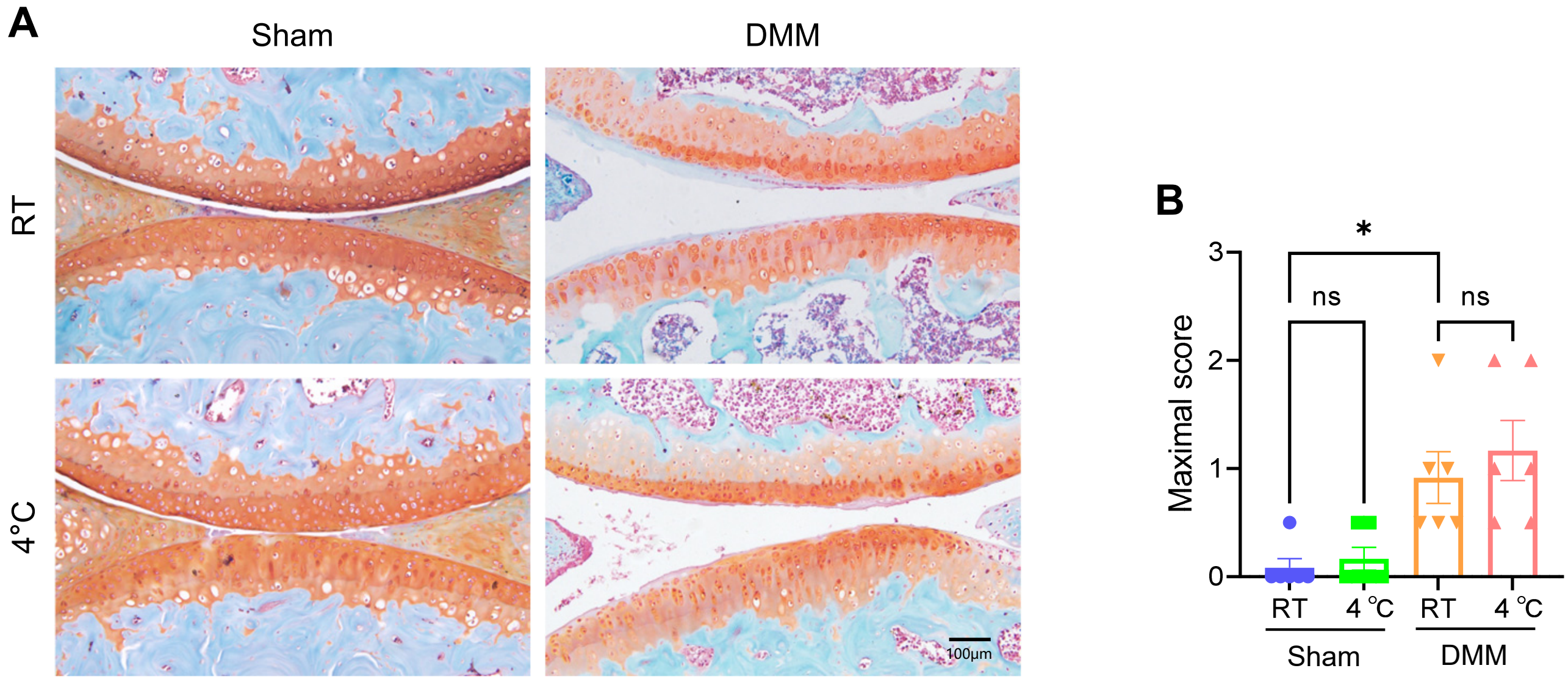
A
Sham
DMM
RT
4°C
100μm
B
*
ns
ns
Maximal score
3
2
1
0
RT
4 ℃
RT
4 ℃
Sham
DMM
