# Supplementary material for: LIPUS promotes calcium oscillations to improve lymphatic drainage and alleviates cold-stimulated pain and effusion in osteoarthritis joints
Source: J Orthop Translat. 2026 Oct 24;61:101242. doi: 10.1016/j.jot.2026.101242 (PMC13721939; doi:10.1016/j.jot.2026.101242)
Supplement: Supplementary file 3 [file mmc3.pdf]

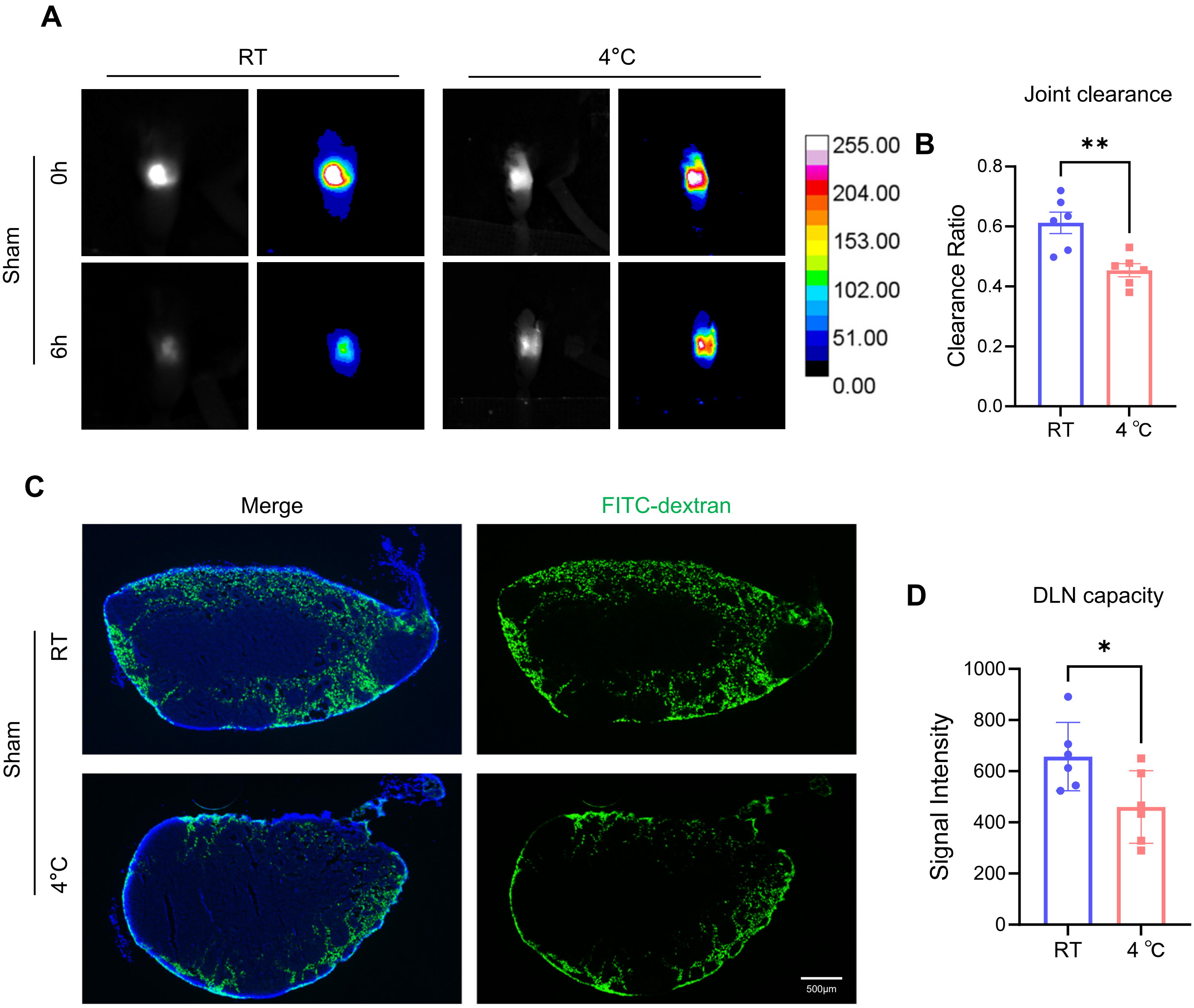

A
RT
4°C
Sham
0h
6h
255.00
204.00
153.00
102.00
51.00
0.00
B
Joint clearance
**
Clearance Ratio
0.8
0.6
0.4
0.2
0.0
RT
4 ℃
C
Merge
FITC-dextran
Sham
RT
4°C
500μm
D
DLN capacity
*
Signal Intensity
1000
800
600
400
200
0
RT
4 ℃
