# Supplementary material for: LIPUS promotes calcium oscillations to improve lymphatic drainage and alleviates cold-stimulated pain and effusion in osteoarthritis joints
Source: J Orthop Translat. 2026 Oct 24;61:101242. doi: 10.1016/j.jot.2026.101242 (PMC13721939; doi:10.1016/j.jot.2026.101242)
Supplement: Supplementary file 4 [file mmc4.pdf]

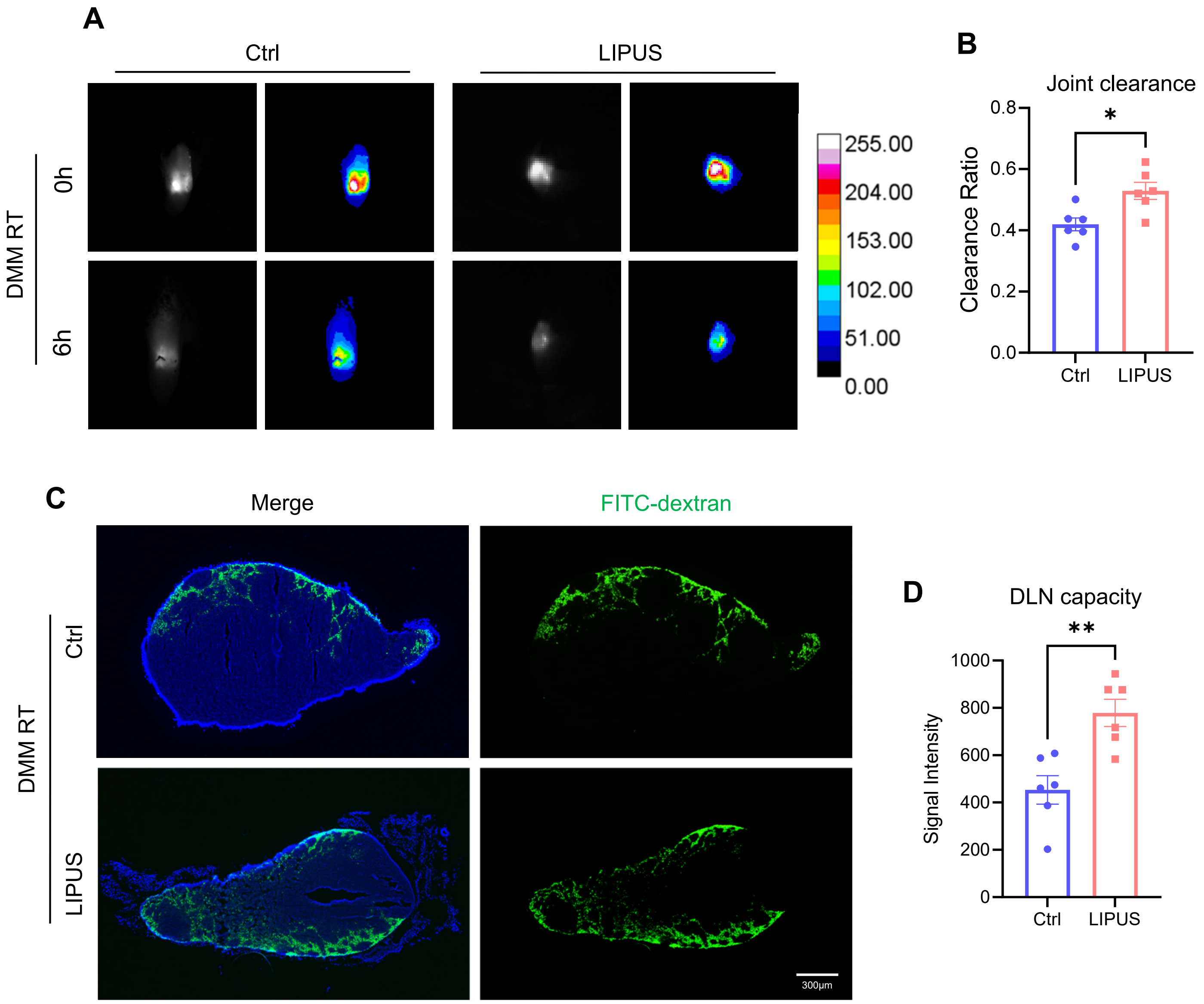

A
Ctrl
LIPUS
DMM RT
0h
6h
255.00
204.00
153.00
102.00
51.00
0.00
B
Joint clearance
Clearance Ratio
0.8
0.6
0.4
0.2
0.0
*
Ctrl
LIPUS
C
Merge
FITC-dextran
DMM RT
Ctrl
LIPUS
300μm
D
DLN capacity
Signal Intensity
1000
800
600
400
200
0
**
Ctrl
LIPUS
