# Supplementary material for: LIPUS promotes calcium oscillations to improve lymphatic drainage and alleviates cold-stimulated pain and effusion in osteoarthritis joints
Source: J Orthop Translat. 2026 Oct 24;61:101242. doi: 10.1016/j.jot.2026.101242 (PMC13721939; doi:10.1016/j.jot.2026.101242)
Supplement: Supplementary file 5 [file mmc5.pdf]

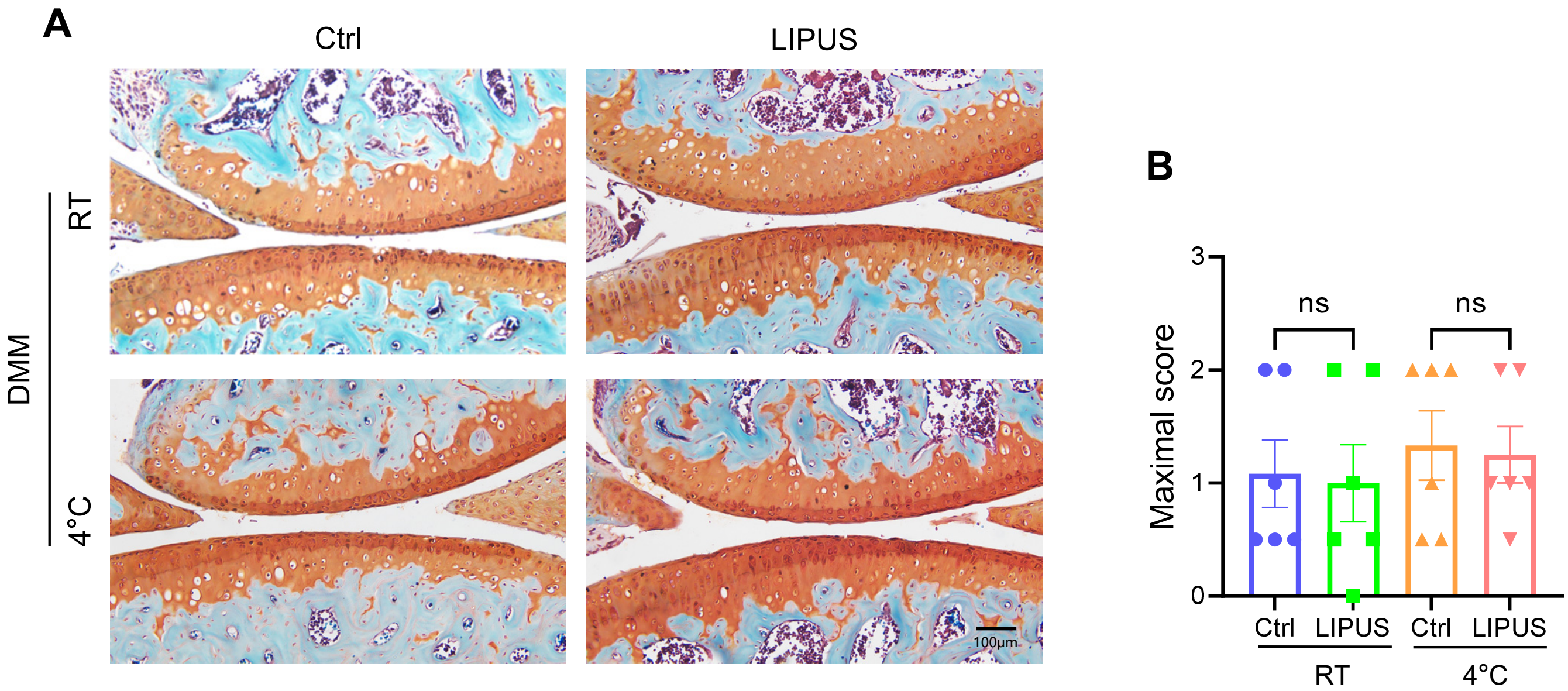
A
Ctrl
LIPUS
DMM
RT
4°C
100μm
B
Maximal score
3
2
1
0
ns
ns
Ctrl
LIPUS
Ctrl
LIPUS
RT
4°C
