# Supplementary material for: LIPUS promotes calcium oscillations to improve lymphatic drainage and alleviates cold-stimulated pain and effusion in osteoarthritis joints
Source: J Orthop Translat. 2026 Oct 24;61:101242. doi: 10.1016/j.jot.2026.101242 (PMC13721939; doi:10.1016/j.jot.2026.101242)
Supplement: Supplementary file 6 [file mmc6.pdf]

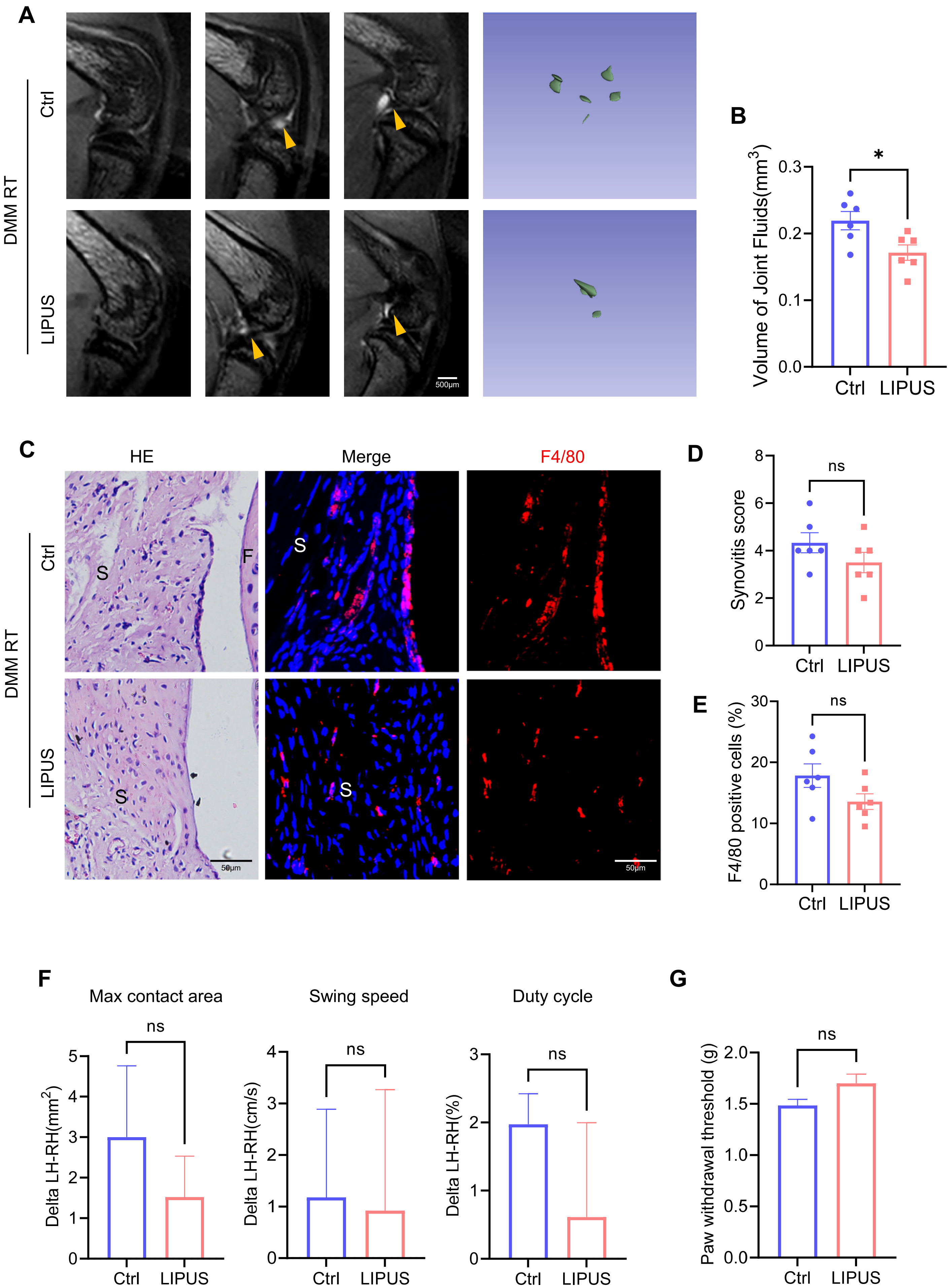
A
DMM RT
Ctrl
LIPUS
500μm
B
Volume of Joint Fluids(mm3)
0.3
0.2
0.1
0.0
*
Ctrl
LIPUS
C
HE
Merge
F4/80
S
F
50μm
D
Synovitis score
8
6
4
2
0
ns
E
F4/80 positive cells (%)
30
20
10
F
Max contact area
Swing speed
Duty cycle
Delta LH-RH(mm2)
Delta LH-RH(cm/s)
Delta LH-RH(%)
5
4
3
2
1
0
G
Paw withdrawal threshold (g)
2.0
1.5
1.0
0.5
0.0
