# Supplementary material for: LIPUS promotes calcium oscillations to improve lymphatic drainage and alleviates cold-stimulated pain and effusion in osteoarthritis joints
Source: J Orthop Translat. 2026 Oct 24;61:101242. doi: 10.1016/j.jot.2026.101242 (PMC13721939; doi:10.1016/j.jot.2026.101242)
Supplement: Supplementary file 7 [file mmc7.pdf]

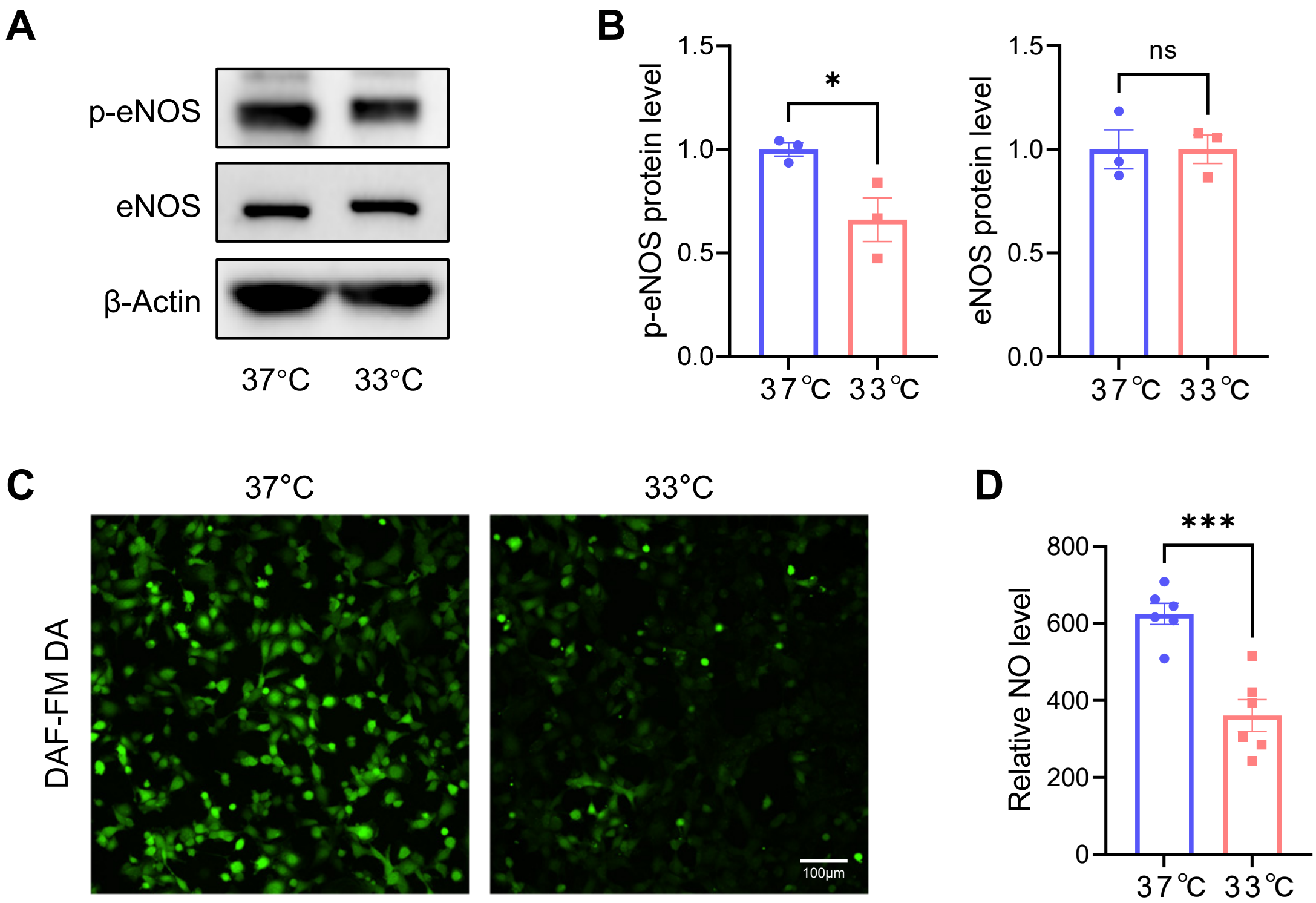

A
p-eNOS
eNOS
β-Actin
37°C
33°C
B
1.5
1.0
0.5
0.0
p-eNOS protein level
*
37℃
33℃
eNOS protein level
ns
C
37°C
33°C
DAF-FM DA
100μm
D
800
600
400
200
0
Relative NO level
***
