# Supplementary material for: LIPUS promotes calcium oscillations to improve lymphatic drainage and alleviates cold-stimulated pain and effusion in osteoarthritis joints
Source: J Orthop Translat. 2026 Oct 24;61:101242. doi: 10.1016/j.jot.2026.101242 (PMC13721939; doi:10.1016/j.jot.2026.101242)
Supplement: Supplementary file 8 [file mmc8.pdf]

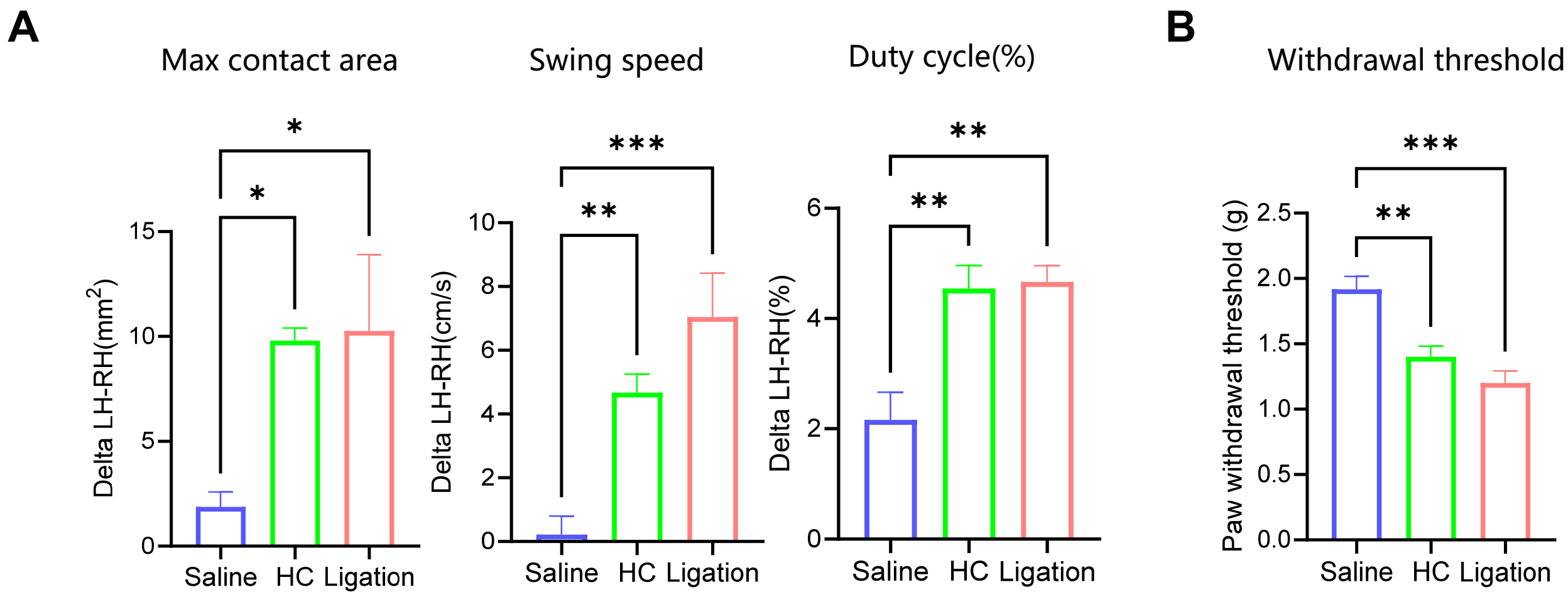

A
Max contact area
Delta LH-RH(mm2)
Saline
HC
Ligation
Swing speed
Delta LH-RH(cm/s)
Duty cycle(%)
Delta LH-RH(%)
B
Withdrawal threshold
Paw withdrawal threshold (g)
